# Organic–Inorganic Triethylenediamine Cu(I)-Iodides as Reusable Photoluminescent Sensors for Waterborne Pollutants

**DOI:** 10.3390/molecules31091384

**Published:** 2026-04-22

**Authors:** Victoria Martín, Giulia Bardelli, Julián Ávila Durán, Pilar Amo-Ochoa

**Affiliations:** 1 Inorganic Chemistry Department, Faculty of Sciences, Autonomous University of Madrid (UAM), 28049 Madrid, Spain; mariav.martin@uam.es (V.M.); giulia.bardelli@estudiante.uam.es (G.B.); julian.avila@estudiante.uam.es (J.Á.D.); 2 Institute for Advanced Research in Chemical Sciences (IAdChem), Autonomous University of Madrid (UAM), 28049 Madrid, Spain

**Keywords:** luminescent Cu(I) halide organic inorganic, nanoparticles, sensor, detection, material processing, tetracycline, iron, water remediation

## Abstract

Luminescent organic–inorganic Cu(I) halide hybrid molecular crystals exhibit remarkable structural diversity and photophysical properties, but their application in aqueous environments is often limited by insufficient stability. Herein, we report portable and reusable photoluminescent sensors based on Cu(I)–I triethylenediamine derivatives [Cu_4_I_6_(pr-ted)_2_] and [Cu_3_I_5_(bz-ted)_2_] (pr-ted = 1-propyl-1,4-diazabicyclo[2.2.2]octan-1-ium; bz-ted = 1-benzyl-1,4-diazabicyclo[2.2.2]octan-1-ium). Their submicrometric particles exhibit intense UV-excited emissions and high photoluminescence quantum yields but limited water stability. To address this limitation, ultrasound sonication was employed to control particle size and produce stable suspensions that can be incorporated into polymeric matrices via 3D printing with photocurable resins or polylactic acid (PLA) films by drop-casting, yielding mechanically robust composites that retain their structural and optical properties. The devices used act as selective turn-off luminescent sensors for Fe^3+^ in aqueous media, with nanomolar detection limits (1.33–1.58 nM) below regulatory thresholds for drinking water. Moreover, [Cu_3_I_5_(bz-ted)_2_] enables tetracycline detection in river water with a limit of detection of 0.038 nM. Mechanistic studies indicate that reversible photoinduced electron transfer is the primary quenching pathway, while composites maintain sensing performance over multiple reuse cycles.

## 1. Introduction

The contamination of water resources by inorganic and organic pollutants has become a major global concern due to its detrimental effects on wildlife and human health, ecosystem integrity, and environmental sustainability [1]. Anthropogenic activities such as industrial discharge, agricultural runoff, mining, and improper waste management continuously introduce hazardous substances into aquatic systems, including heavy metal ions and persistent pharmaceutical residues [2,3]. Among them, iron and antibiotics such as tetracycline are particularly relevant: while Fe^3+^ is an essential element for biological processes, excessive intake leads to pathological disorders associated with iron accumulation, prompting strict regulatory limits in drinking water [4,5,6]. In parallel, tetracycline is frequently detected in surface and wastewater due to its high stability and poor biodegradability, raising serious concerns regarding antimicrobial resistance [7].

Conventional analytical techniques for water quality monitoring, including high-performance liquid chromatography [8], gas chromatography coupled with mass spectrometry [8,9], and Raman spectroscopy [10], provide excellent sensitivity and accuracy. However, their reliance on sophisticated instrumentation, high operational costs, and limited portability restricts their use for on-site and real-time analysis. In response to these limitations, luminescence-based chemical sensors have emerged as powerful alternatives, offering rapid response, low detection limits, operational simplicity, and potential for miniaturization [11]. In particular, photoluminescent sensors operating through reversible mechanisms are especially attractive, as they enable multiple sensing cycles without permanent degradation of the active material [12,13]. A wide variety of luminescent materials have been explored for aqueous sensing applications, including semiconductor quantum dots [14], lanthanide-based coordination compounds [15], carbon-based nanomaterials, and metal–organic materials ranging from discrete coordination complexes to extended metal–organic frameworks (MOFs) [16,17,18]. Each class presents specific advantages and limitations in terms of emission efficiency, chemical stability, and processability. Among them, Cu(I) halide coordination compounds have attracted increasing attention due to their rich structural diversity and photophysical nature [19]. In many luminescent organic–inorganic Cu(I) halide coordination compounds, photoluminescence typically originates from a variety of excited states, primarily triplet cluster-centered (CC) states and metal/halide-to-ligand charge transfer (M/XLCT) processes [20]. The relative energies and intensities of these emissive states are highly sensitive to structural parameters such as Cu···Cu distances, halide identity, and the ligand environment, which effectively modulate the electronic structure and the thermal energy barriers of Cu–halide systems [21,22].

Despite these attractive features, many Cu(I) halide molecular systems suffer from limited long-term stability in aqueous media, which hinders their direct application as water-compatible sensors [23,24,25]. To address this challenge, different strategies have been proposed, including encapsulation in polymeric matrices, surface immobilization, and processing into composite materials, which can significantly enhance chemical robustness while preserving luminescent performance [26,27]. In particular, the integration of luminescent coordination compounds into polymer supports enables the development of mechanically stable, portable, and reusable sensing platforms. In this context, two previously reported organic–inorganic Cu(I)–iodide compounds based on triethylenediamine derivatives, [Cu_4_I_6_(pr-ted)_2_] and [Cu_3_I_5_(bz-ted)_2_], were selected due to their intense visible emission, near-unity photoluminescence quantum yields, and remarkable structural stability across a broad pH range [28,29]. These compounds were processed into portable photoluminescent platforms by incorporating them into 3D-printed photocurable resins and polylactic acid (PLA) films, yielding composite materials that can operate in aqueous environments while maintaining their optical response.

Herein, we report a comprehensive study of the photophysical properties, aqueous stability, and sensing performance of these Cu(I)–Iodine compounds. In addition, we used ultrasound and ball milling to control their particle size, forming stable suspensions that enable the creation of useful composite materials for environmentally relevant pollutants. The resulting systems function as highly selective and sensitive turn-off luminescent sensors for Fe(III) ions and, in the case of [Cu_3_I_5_(bz-ted)_2_], for tetracycline, operating through a reversible photoinduced electron transfer (PET) mechanism. By combining Cu(I) Iodine cluster chemistry with polymer-based processing, this work explores a versatile and robust strategy for developing reusable luminescent sensors with direct relevance to water quality monitoring [6].


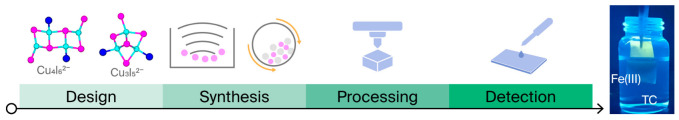


## 2. Results and Discussion

### 2.1. Chemical, Morphological and Optical Characterization of [Cu_3_I_5_(bz-ted)_2_] and [Cu_4_I_6_(pr-ted)_2_] Particles

Initially, both compounds were synthesized under mild conditions using poly(vinylpyrrolidone) (PVP) as a capping agent to control particle size [30,31]. First, PVP was dissolved in ethanol. Separately, CuI was dissolved in a potassium-iodide-saturated aqueous solution. The CuI solution was added dropwise to the PVP solution, forming a homogeneous yellow suspension. Subsequently, a solution of the corresponding ligand in ethanol was added, and the mixture was stirred at room temperature [27].

**Figure 1:**
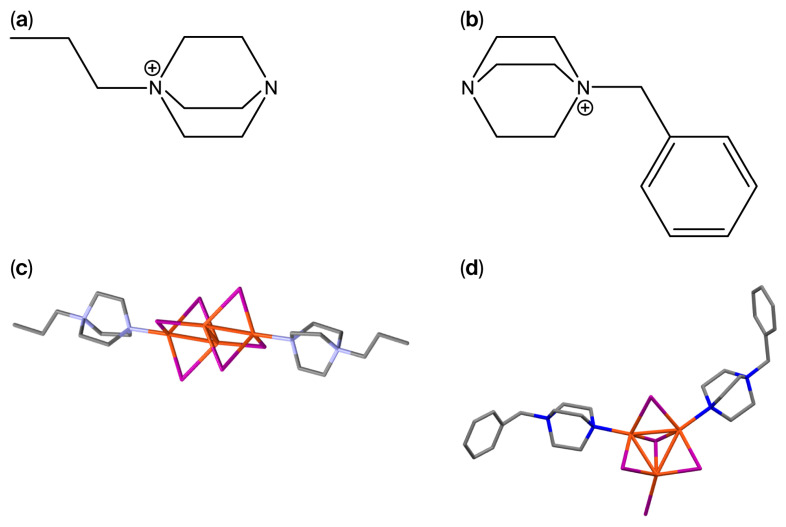
Schematic representation of the organic ligands 1-Propyl-1,4-diazabicyclo[2.2.2]octan-1-ium (pr-ted) (**a**) and 1-benzyl-1,4-diazabicyclo[2.2.2]octan-1-ium (bz-ted) (**b**). Fragments of crystal structures of [Cu_4_I6(pr-ted)_2_] (**c**), and [Cu_3_I_5_(bz-ted)_2_] (**d**). Hydrogens have been omitted for clarification. Blue represents nitrogen atoms; violet represents Iodine atoms; grey represents carbon atoms; and orange represents copper atoms.

The purity of the products was confirmed by elemental analysis, with experimental values in good agreement with the calculated compositions. FTIR spectra display the characteristic vibrational bands of the pr-ted and bz-ted ligands. Upon coordination to Cu(I), the ν(C–N) stretching vibration shows a slight shift from 1057 and 1053 cm^−1^ in the free bz-ted and pr-ted ligands, respectively, to 1049 for [Cu_3_I_5_(bz-ted)_2_] and 1047 cm^−1^ for [Cu_4_I_6_(pr-ted)_2_] in the corresponding complexes. Additionally, the ν(N–H) stretching bands observed at 3422 cm^−1^ for bz-ted and 3410 cm^−1^ for pr-ted disappear after complex formation. These bands are associated with the N-H stretching vibrations of the triethylenediamine derivatives in their salt/partially protonated form used during the synthesis. Their disappearance confirms the successful coordination of the nitrogen atoms to the Cu(I) centers, which modifies the electronic environment of the ligand. The C–H stretching vibrations remain essentially unchanged, confirming preservation of the organic ligand frameworks. Bands at 1676 cm^−1^ for [Cu_3_I_5_(bz-ted)_2_] and 1699 cm^−1^ for [Cu_4_I_6_(pr-ted)_2_] are assigned to the C=O stretching vibration of residual PVP used during synthesis (Figures S3 and S5). The presence of this polymer does not affect the properties of the final materials. The structures of the compounds have been previously described [28,29] in the form of two types of Cu-I clusters, [Cu_4_I_6_]^2−^, and [Cu_3_I_5_]^2–^, with short Cu–Cu distances of 2.521–2.695 Å and 2.620–2.748 Å, and Cu–I distances of 2.366–2.649 Å and 2.578–2.672 Å, respectively, and tetrahedral environments for each CuI_3_N (Figure 1).

Powder X-ray diffraction (PXRD) patterns of [Cu_3_I_5_(bz-ted)_2_] and [Cu_4_I_6_(pr-ted)_2_] closely match the simulated patterns derived from single-crystal X-ray diffraction data, confirming that the crystal structures are preserved in synthesized particles (Figures S4–S6).

**Figure 2:**
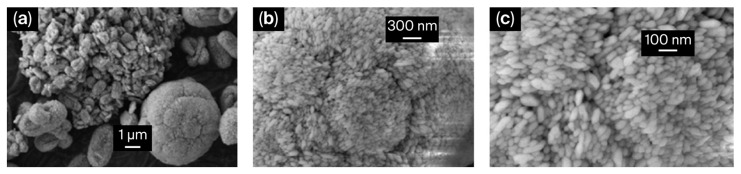
SEM image of [Cu_3_I_5_(bz-ted)_2_] at ×5000 magnification (**a**); SEM image at ×25,000 magnification (**b**); and SEM image at ×50,000 magnification (**c**).

Morphological characterization in the solid state was carried out using scanning electron microscopy (SEM). Although the SEM images of [Cu_4_I_6_(pr-ted)_2_] show spherical submicrometric particles with an average diameter of 369.2 ± 129.5 nm [27], [Cu_3_I_5_(bz-ted)_2_] SEM images reveal spherical-to-oval particles with a rice-grain-like morphology. Statistical analysis of 30 particles resulted in average dimensions of 117.5 ± 36.8 nm in length and 54.4 ± 16.1 nm in width (Figure 2, Figures S7 and S8).

**Figure 3:**
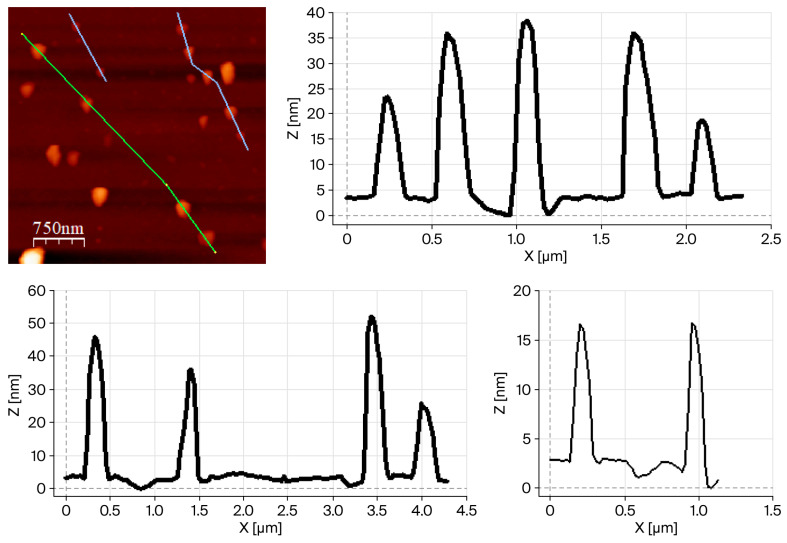
AFM image of [Cu_3_I_5_(bz-ted)_2_] particles obtained after 10 min of sonication (ultrasonic bath; 80% power in ethanol) with the corresponding height and width profiles.

To reduce the apparent particle size in suspension, the isolated PVP-stabilized compounds were subjected to ultrasonic treatment (ultrasonic bath, 25 °C, and 80% power) for 10 min in water and ethanol. After sonication, both materials were re-characterized using FTIR spectroscopy and PXRD, which confirmed that no phase transformation or chemical modification occurred during the treatment (Figures S9–S12). The ethanol suspensions of [Cu_3_I_5_(bz-ted)_2_] obtained after 10 min of sonication were further analyzed using atomic force microscopy (AFM), confirming a reduction in particle size. Under these conditions (80% sonication power), statistical analysis of the particles (n = 30) yielded an average particle width of 166 ± 48 nm and an average height of 25 ± 10 nm, which are in close agreement with the individual particles observed using the SEM. (Figure 3 and Figures S13–S15).

Additionally, to improve the sustainability of the synthetic procedure and reduce the particle size to create stable suspensions, a solvent-free reaction was performed using the ball milling approach. Elemental analysis, IR spectra, and DRXP show the expected [Cu_3_I_5_(bz-ted)_2_] (Figures S16 and S17). SEM images of [Cu_3_I_5_(bz-ted)_2_] obtained by ball milling reveal spherical-to-oval particles. Statistical analysis of 30 particles yields an average particle diameter of 138 ± 25 nm (n = 30) (Figures S18 and S19). This approach is significant due to its scaling of the synthesis and use of nanoparticles in the processing of sensor devices.

### 2.2. Photoluminescence Properties

**Figure 4:**
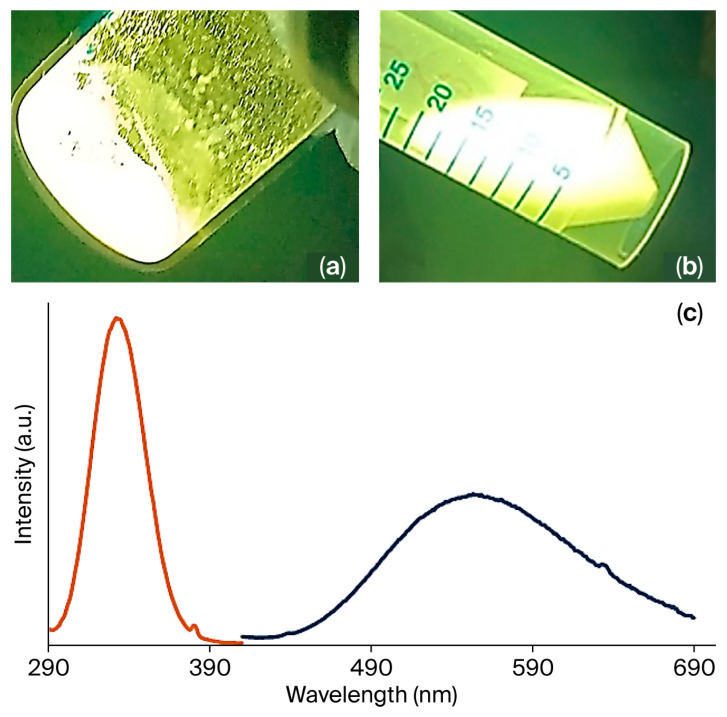
[Cu_3_I_5_(bz-ted)_2_] emissions under UV light (λ_ex_ = 365 nm) in solid form (**a**) and suspended in ethanol (**b**). Excitation (orange) (λ_em_ = 555 nm) and emission (dark blue) (λ_ex_ = 355 nm) spectra of [Cu_3_I_5_(bz-ted)_2_] in solid state (**c**).

The Cu(I)–iodide coordination compounds [Cu_4_I_6_(pr-ted)_2_], [Cu_3_I_5_(bz-ted)_2_] exhibit intense visible photoluminescence under UV irradiation (365 nm), appearing green and orange-yellow, respectively (Figure 4 and Figure S20). This emission is characteristic of cluster-centered excited states commonly observed in Cu(I) halide aggregates with short Cu···Cu separations, as reported for related hybrid Cu(I)–iodide luminophores [32]. Emissions are observed in the solid state and in ethanol and water suspensions (Figure 4a,b). The solid-state photophysical properties were investigated at room temperature for [Cu_3_I_5_(bz-ted)_2_]. The excitation spectrum displays a broad band from 300 to 390 nm with a maximum at 335 nm, while the emission spectrum spans 450–700 nm with a maximum at 555 nm (Figure 4c).

[Cu_4_I_6_(pr-ted)_2_] exhibits a strong emission band centered at 526 nm, which is in agreement with reports in the literature [28] (Figure S21). Upon UV excitation, electrons are promoted to singlet excited states, followed by efficient intersystem crossing to triplet cluster-centered states. Radiative decay from these triplet states accounts for the large Stokes shifts and intense emissions observed. Cu···Cu interactions within the cluster core, together with coordination by the ted ligands, stabilize the triplet excited state and enhance luminescence efficiency [33]. The photoluminescence quantum yield is close to unity for [Cu_4_I_6_(pr-ted)_2_] and approximately 32.6% for [Cu_3_I_5_(bz-ted)_2_].

Preservation of emission profiles upon processing into submicrometric and nanoparticles (Figures S21 and S22) indicates that the cluster-centered electronic structure remains dominant, which is consistent with other reports on solid-state Cu(I) luminescent materials showing minimal efficiency loss upon size reduction [34].

The strong emissions of these types of compounds make them interesting for sensing applications; for this reason, their real stability in water with pH and time was studied. Our research group has recently published findings that although [Cu_4_I_6_(bz-ted)_2_] submicrometric particles are stable in a wide pH range (4–9), they lose this stability in water over time, limiting their long-term use as aqueous sensors [27]. For this reason, and following the same steps, the time-dependent stability of [Cu_3_I_5_(bz-ted)_2_] was assessed using ^1^H NMR spectroscopy. Immediately after preparation, the aqueous suspension shows no NMR signals corresponding to the ligand; however, after 2 h, resonances of the free bz-ted ligand appear, indicating partial decomposition in water (Figure S23). Additionally, pH-dependent stability was examined between pH 2 and 8. PXRD patterns of solids recovered after exposure to acidic and basic conditions closely match those of the pristine material, demonstrating preservation of the crystalline phase (Figure S24). Overall, [Cu_3_I_5_(bz-ted)_2_] shows good structural stability in aqueous media between pH~2.5 and 7.5, although prolonged exposure leads to partial degradation.

**Figure 5:**
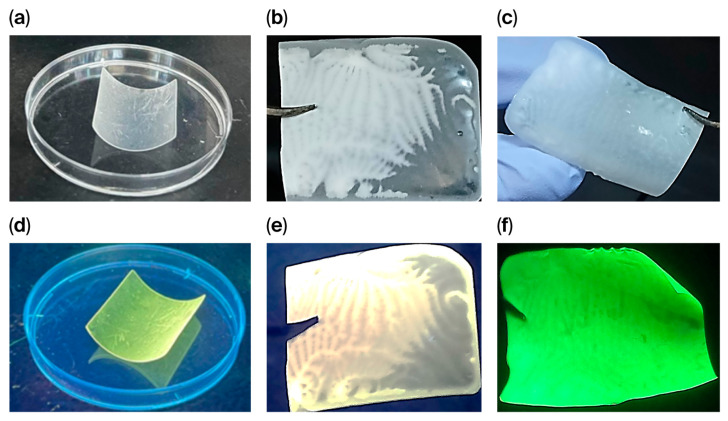
[Cu_3_I_5_(bz-ted)_2_]@3D0.24% under visible light (**a**) and UV light (λₑₓ = 365 nm) (**d**). [Cu_3_I_5_(bz-ted)_2_]@PLA3.6% under visible (**b**) and UV light (λₑₓ = 365 nm) (**e**). [Cu_4_I_6_(pr-ted)_2_]@PLA3.6% under visible light (λₑₓ = 365 nm) (**c**) and [Cu_4_I_6_(pr-ted)_2_]@PLA3.6% UV light [Cu_4_I_6_(pr-ted)_2_]@PLA3.6% (**f**).

Owing to the controlled reduction in nanoparticle size and the formation of stable suspensions using ultrasounds and ball milling, [Cu_3_I_5_(bz-ted)_2_] nanoparticles can be processed using different approaches to overcome their limited aqueous stability. Indeed, we processed powders using different methods. One option is their incorporation into a photopolymerizable resin to produce a 3D-printed composite [Cu_3_I_5_(bz-ted)_2_]@3D0.24% (Figure 5a,d). Another method is the use of drop-casting to create PLA composites (Figure 5b,c,e,f).

Additionally, for IR spectra characterization (Figure S25), PXRD analysis of [Cu_3_I_5_(bz-ted)_2_]@3D0.24% (Figure S27) shows a predominantly amorphous pattern from the resin, together with characteristic diffraction peaks at 10.0°, 11.8°, 21.3°, and 23.6° corresponding to the crystalline phase of the cluster. This confirms the retention of structural integrity. The composite displays emissions under UV irradiation with no visible color change. The excitation and emission maxima (327 and 522 nm, respectively) are slightly blue-shifted compared to the pristine particles (335 and 555 nm), which is attributed to the different dielectric environment of the polymer matrix rather than structural modification (Figure S29). Similarly, the PXRD of [Cu_3_I_5_(bz-ted)_2_]@PLA3.6% and [Cu_4_I_6_(pr-ted)_2_]@PLA3.6% exhibit an amorphous pattern from the PLA, together with the characteristic diffraction peaks of both compounds, confirming the retention of structural integrity (Figures S32 and S33). Moreover, [Cu_3_I_5_(bz-ted)_2_]@PLA3.6% SEM-EDX images show a homogeneous distribution of [Cu_3_I_5_(bz-ted)_2_] particles (Figure S34). ^1^H NMR spectra of [Cu_3_I_5_(bz-ted)_2_]@3D0.24% immersed in D_2_O at *t* = 0 and after 2 h (Figure S35) show only a solvent signal, demonstrating that polymer encapsulation effectively suppresses ligand release and enhances aqueous stability.

### 2.3. Sensing Performance

**Figure 6:**
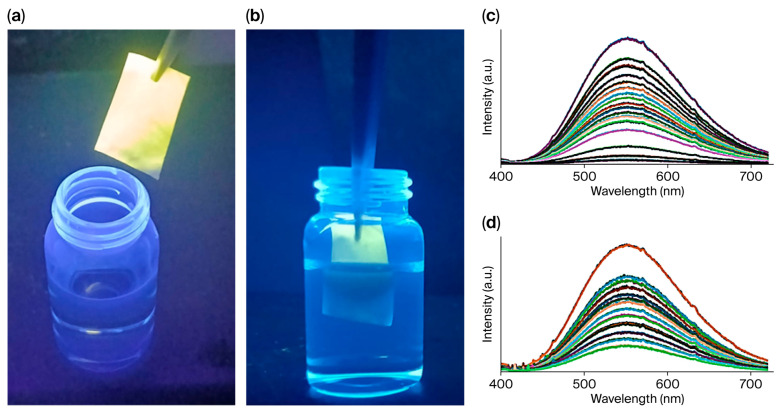
[Cu_3_I_5_(bz-ted)_2_]@PLA3.6% under UV light (**a**), and partially immersed in a 0.01 M Fe (III) solution (**b**). Emission spectra of an aqueous suspension of submicrometric particles of [Cu_3_I_5_(bz-ted)_2_] with respect to the Fe(III) concentration in river water (**c**). Emission spectra of an aqueous suspension of submicrometric particles of [Cu_3_I_5_(bz-ted)_2_] with respect to the TC concentration in river water (**d**).

The sensing performance toward Fe(III), both in laboratory and river water, shows pronounced and selective luminescence quenching for both Cu(I)–iodide systems, with limits of detection (LOD) in the low nanomolar range (1.33–1.58 nM) (Figure 6, Figures S36 and S37). These values compare favorably with those reported for coordination polymers and MOFs, which typically operate in the low-to-mid nanomolar regime [35].

A notable distinction is observed for tetracycline (TC) sensing in laboratory and river waters. While [Cu_4_I_6_(pr-ted)_2_] shows limited sensitivity, [Cu_3_I_5_(bz-ted)_2_] exhibits highly sensitive detection with an LOD of 0.038 nM in river water, significantly outperforming many reported luminescent sensors that operate in the micromolar or high nanomolar range (Figures S38 and S39 and Table 1) [36,37,38,39,40].

Fluorescence quenching can originate from different processes. One possible process involves a chemical reaction between the luminescent compound and the analyte.

To investigate whether a chemical reaction occurs, a previously reported procedure was followed for both [Cu_3_I_5_(bz-ted)_2_] and [Cu_4_I_6_(pr-ted)_2_]. In this method, the luminescent compound and the Fe(III) compound were mixed in deionized water. Subsequently, the solid was separated from the solution, and the X-ray diffractogram of the recovered material was recorded and compared with that of the pristine compound. In these cases, no significant structural changes were observed, indicating that no chemical reaction occurred that could compromise the sensor’s reusability (Figure S40). A reversible quenching mechanism is generally preferred for sensing applications, as it allows the sensor to be reused multiple times. Another commonly proposed mechanism for emission quenching is photoinduced electron transfer (PET), in which the excited-state electron of the luminescent compound is transferred to the analyte, resulting in non-radiative relaxation and a decrease in emissions [47,48,49]. This process occurs when the energy level of the LUMO orbital in the luminescent material is higher than that of the analyte. Under these conditions, an electron can be transferred from the luminescent material to the analyte, resulting in fluorescence quenching. In other words, an electron transfer from the luminescent compound (donor) to the analyte (acceptor) prevents the excited electron from returning radiatively to the ground state. Other possible pathways, such as the inner filter effect (IFE), may also contribute to emission quenching depending on the optical properties of both the analyte and the luminescent compound. The IFE can be experimentally verified by comparing the UV–Vis absorption spectra of the luminescent compound, the analyte, and their mixture. If the analyte shows significant absorption at the excitation and/or emission wavelengths of the compound, the apparent fluorescence quenching may arise from the IFE rather than from a genuine electronic interaction between the two species (Figure S41) [50]

Interference studies were carried out on [Cu_3_I_5_(bz-ted)_2_] and [Cu_4_I_6_(pr-ted)_2_] particles using various metal ions (Al(III), Ca(II), Cr(III), Cu(II), Pb(II), Mg(II), and Na(I)). These experiments were performed by adding 200 µL of 0.01 M solutions of the corresponding ions. In addition, the influence of other antibiotics, such as sulfonamide and chloramphenicol, was also evaluated. The results indicate that, in the case of [Cu_3_I_5_(bz-ted)_2_], the presence of Pb(II) can slightly interfere with the detection of Fe(III) at a concentration of 10%. In contrast, for [Cu_4_I_6_(pr-ted)_2_], cations such as Na(I), Mg(II), Ca(II), Al(III), Pb(II), and Cr(III), a minor interference effect may be produced. However, no interference from other antibiotics was observed in the detection of TC for either of the two compounds (Figures S42–S45).

It should be emphasized that devices fabricated by incorporating these materials into PLA composites using 3D printing have already demonstrated high stability during the detection of TC [27]. The new devices prepared with [Cu_3_I_5_(bz-ted)_2_]@3D0.24% and [Cu_3_I_5_(bz-ted)_2_]@PLA3.6%, as well as [Cu_4_I_6_(pr-ted)_2_]@PLA3.6%, show excellent stability toward TC and Fe(III) detection over a large number of measurements. Powder X-ray diffraction analyses of [Cu_3_I_5_(bz-ted)_2_]@3D0.24% confirm that the crystalline structure remains stable after 20 immersion cycles in solutions containing either TC or Fe(III) (Figures S46 and S47).

## 3. Materials and Methods

All chemicals and reagents were used as purchased from commercial suppliers. Triethylenediamine (≥99%), 1-bromopropane (≥99%), polyvinylpyrrolidone (PVP, CAS: 9003-39-8), cuprous iodide (≥98%), and copper (I) bromide were supplied by Sigma Aldrich (Burlington, MA, USA). Copper (I) chloride (97+%) was supplied by Thermo Scientific (Waltham, MA, USA), and ethanol (≥99.9%) was purchased from Scharlau (Barcelona, Spain). The supplier for acetone (≥99.8%) is Carlo Erba (Val-de-Reuil, France). KI (≥99%) was supplied by Labkem (Dublin, Ireland); benzyl bromide was supplied by Sigma Aldrich; and ethyl acetate was obtained from Scharlau. The metal nitrates used in this work are iron (III) nitrate nonahydrate (98%), magnesium nitrate hexahydrate (99%), copper (II) nitrate trihydrate, aluminum nitrate nonahydrate (98%), and sodium nitrate from Aldrich; calcium nitrate tetrahydrate from Scharlau; chromium (III) nitrate nonahydrate from Panreac (Barcelona, Spain); and lead (II) nitrate from Fisher. The antibiotics used in this work are sulfamethazine (SMZ, 99.0–101.0%, CAS: 57-68-1), chloramphenicol (CAP, 98%, CAS: 56-75-7), and tetracycline (TC, 98.0–102.0%, CAS: 60-54-8), all purchased at Sigma Aldrich. Polylactic acid–biopolymer compounds (PLA) and granules (3 mm in nominal granule size) were obtained from Aldrich. Standard Photopolymer Translucid Resin was purchased from ELEGOO (Lewes, DE, USA).

Elemental Analyses (EAs) were performed on a LECO CHNS-932 elemental analyzer.

Powder X-ray Diffraction (PXRD) data were collected on a Rigaku Miniflex 600 HyPix-400 MF 2D (Tokyo, Japan) diffractometer equipped with a 600 W X-ray source (Cu-Kα radiation; λ = 1.5418 Å) in a θ/2θ geometry. Samples were scanned over a 2θ range of 3–50°, with an angular increment of 0.03° and a counting time of 10° per minute.

Infrared (FTIR-ATR) spectra were recorded on a PerkinElmer spectrophotometer (Shelton, CT, USA) equipped with a MIRacle Universal Attenuated Total Reflectance (ATR) accessory.

A Hitachi S-3000N scanning electron microscopy (SEM-EDX) microscope (Tokyo, Japan) operating at an accelerating voltage of 5.0 kV and a chamber pressure of 10^−9^ Pa, was used to study the morphology and average size of the [Cu_3_I_5_(bz-ted)_2_] and [Cu_4_I_6_(pr-ted)_2_] particles, which were both as-obtained and sonicated for 10 min (80% power; 25 °C). Samples were coated with a thin chromium layer by sputtering before imaging. Additionally, the spatial distribution of the [Cu_3_I_5_(bz-ted)_2_]@PLA composite was evaluated using SEM-EDX mapping.

An Elma Transsonic Digital S ultrasonic (US) bath (Singen, Germany) and a VASCO Particle Size Analyzer (Cordouan Technologies) (Pessac, France) were used. Suspensions containing 5 mg of [Cu_3_I_5_(bz-ted)_2_] and [Cu_4_I_6_(pr-ted)_2_], respectively, in 5 mL of ethanol were prepared under magnetic stirring (30 min, 1000 rpm). The resulting suspensions were then sonicated in an ultrasonic bath for 10 min (80% power) at room temperature.

Atomic force microscopy (AFM) measurements were carried out using a Cervantes Fullmode AFM from Nanotec Electronica SL. WSxM v5.0 software (20 April 2026, https://www.wsxmsolutions.com) was used for both data acquisition and image processing. PPP-NCHR cantilevers (20 April 2026, https://www.nanosensors.com) with a nominal spring constant of 42 N m^−1^ and tip radius of less than 7 nm were employed.

First, SiO_2_ wafers were sliced into square pieces of approximately 0.5 × 0.5 cm. These square wafers were placed onto a vial and sonicated for 15 min in acetone in an ultrasound bath (80% power; room temperature), followed by 15 min of sonication (80% power; room temperature) in isopropanol. After sonication, they were removed from the vial, and isopropanol was allowed to evaporate.

Finally, a suspension of [Cu_3_I_5_(bz-ted)_2_] 0.33 mg/mL in ethanol was prepared. The mixture was placed under magnetic stirring (10 min, room temperature, and 800 rpm) and later sonicated in an ultrasound bath at room temperature with 80% power for 10 min. An aliquot of 5 µL of the resulting suspension was deposited onto a SiO_2_ substrate by drop-casting, and ethanol was allowed to evaporate under ambient conditions prior to AFM analysis.

Nuclear magnetic resonance (^1^H-NMR) spectra were recorded using a BRUKER AVIII HD-300MHz spectrometer (Karlsruhe, Germany).

For fluorescence spectroscopy, the luminescent properties of [Cu_3_I_5_(bz-ted)_2_], [Cu_3_I_5_(bz-ted)_2_]@3D_0.24_%, and [Cu_4_I_6_(pr-ted)_2_] were studied using a FS5 spectrofluorometer (Edinburgh Instruments) equipped with a xenon arc lamp as the excitation source. The instrument was also used to perform luminescence-based sensing experiments to evaluate the detection capabilities of both coordination compounds toward pollutants in water.

An ELEGOO Mars 2 Pro 3D printer (Shenzhen, China) was used. The software used for designing the models was Tinkercad. An ELEGOO light-curing translucent resin (solidification wavelength = 405 nm; hardness = 84; viscosity = 150–200 Mpas; liquid density = 1.100 g/cm^3^; solid density = 1.195 g/cm^3^; flexural strength = 59–70 Mpa; and tensile strength = 36–53 MPa) was used to generate the composites. The solvent used to clean the composites was isopropanol. After 3D printing, the device was placed in an ELEGOO Mercury Plus curing station for 2 min, one minute on each side, to achieve post-curing to ensure complete solidification of the resin.

Synthesis of the triethylenediamine (ted) benzyl (bz-ted), propyl (pr-ted), [Cu_3_I_5_(bz-ted)_2_], and [Cu_4_I_6_(pr-ted)_2_].

Synthesis of 1-Benzyl-1,4-diazabicyclo[2.2.2]octan-1-ium (bz-ted)

The synthesis was carried out as already reported by Liu et al. [29]: yield: 85%; elemental analysis (cal C_13_H_19_N_2_): C: 76.73%; H: 9.34%; and N: 13.7%; found: C: 76.74%; H: 9.38%; and N: 13.76%. δ ^1^H NMR (300 MHz, D_2_O): δ 7.44–7.55 (m, 5H, Ar–H), 4.44 (s, 2H, CH_2_–Ph), 3.12 (m, 6H, DABCO CH_2_), and 3.40 (m, 6H, DABCO CH_2_) (Figure S1).

FT-IR (cm^−1^): 3422 w (N-H stretching); 2960 w (=C-H stretching); 1454 m (C-H(-CH_2_-) Bending); 1057 m (C-N stretching); 700 m and 763 m (C=C-H Bending).

Synthesis of the 1-propyl-1,4-diazabicyclo[2.2.2]octan-1-ium (pr-ted)

The synthesis was carried out as reported by E. de la Rubia et al. [27]: Yield 91%. ^1^H-NMR (300 MHz, D_2_O): δ 3.93 (m, 6H, DABCO CH_2_), 3.14 (m, 6H, DABCO CH_2_), 3.30 (m, 2H, CH_2_–N+ propil), 1.71 (m, 2H, CH_2_ central propil), and 0.90 (t, 3H, CH_3_ terminal propil) (Figure S2). IR (cm^−1^): 3410 (b), 2961 (m), 2882 (m), 1633 (w), 1464 (m), 1418 (w), 1379 (w), 1325 (w), 1194 (w), 1094 (s), 1053 (s), 986 (s), 943 (m), 888 (m), 843 (s), 795 (m), 747 (w), and 696 (m). Calculated elemental analysis: C, 45.97%; H, 8.14%; and N, 11.91%. Found: C, 45.21%; H, 8.24%; and N, 11.64%.

Synthesis of [Cu_3_I_5_(bz-ted)_2_] by PVP

On the one hand, 0.25 g of PVP was dissolved at room temperature in 12.5 mL of ethanol by magnetic stirring (600 rpm, 1 h). A colorless solution was observed. On the other hand, 2 g of KI was dissolved in 2 mL of deionized water. Over the KI solution, 0.097 g (0.5 mmol) of CuI was added. When CuI was completely dissolved, a yellowish solution was observed. In total, 0.1 g (0.5 mmol) of bz-ted was dissolved in 0.5 mL of ethanol, obtaining a colorless solution. The CuI solution was added dropwise to the PVP one, obtaining a yellow homogeneous suspension. Afterwards, the bz-ted solution was added dropwise to the suspension. The mixture was left at room temperature under magnetic stirring for 45 min (600 rpm), and a yellowish suspension formed. The suspension was placed under centrifugation for 30 min (6000 rpm), and a white precipitate formed. The yellow supernatant was removed, and the obtained powder was washed once with deionized water and once with ethanol. After washing it, it was then dried under vacuum for 24 h. A yield of 95.7% was obtained for this reaction. Elemental analysis (cal C_3_I_5_C_16_H_38_N_4_): C: 25.35%; H: 3.11%; and N: 4.55%. Exp.: C: 25.92%; H: 3.61%; and N: 4.71%. FT-IR (cm^−1^): 2954m (=C-H Str), 1676s (C=O, Str), 1453m (C-H(-CH_2_-) Bend), 1049s (C-N Str), and 711 and 768m (C=C-H Bend).

Synthesis of [Cu_4_I_6_(pr-ted)_2_] by PVP

The syntheses of pr-ted and [Cu_4_I_6_(pr-ted)_2_] were carried out as already reported by De la Rubia et al. [27]. This form of synthesis is an optimized method of Jing-Jing Wang et al.’s previously reported method [28]. Elemental analysis calculated for [Cu_4_I_6_(pr-ted)_2_](PVP)_0.6_: C, 18.65%; H, 3.14%; and N, 4.63%. Found: C, 18.4%; H, 3.43%; and N, 4.45%. FT-IR (cm^−1^): 2996 (w), 2970 (w), 1456 (s), 1375 (s), 1318 (s), 1096 (m), 1048 (m), 1004 (s), 843 (s), 795 (s), 759 (m), and 708 (m) (Figure S6). Yield: 76.5%.

Solvent-free synthesis of [Cu_3_I_5_(bz-ted)_2_] by ball milling

In total, 0.097 g of CuI (0.5 mmol) was mixed with 0.1 g of bz-ted (0.5 mmol) onto an IKA Tube ST-20-M. Thirty stainless-steel balls (5 mm) were added to the tube, and the mechanochemical synthesis was carried out by ball milling using an IKA Ultra Turrax (Power: 8) for 29 min. The obtained products were washed with ethanol by centrifugation (6000 rpm, 30 min). Elemental analysis (cal Cu_3_I_5_C_16_H_38_N_4_): C: 25.35%; H: 3.11; %, and N: 4.55%. Exp.: C 28.7%, H 3.8%, N 4.85% FT-IR (cm^−1^): 2954m (=C-H Str), 1676s (C=O, Str), 1453m (C-H(-CH_2_-) Bend), 1049s (C-N Str), and 711 and 768m (C=C-H Bend). Yield: 76%.

Utrasounds (US) of [Cu_3_I_5_(bz-ted)_2_] and [Cu_4_I_6_(pr-ted)_2_].

In total, 10 mg of compounds [Cu_3_I_5_(bz-ted)_2_] and [Cu_4_I_6_(pr-ted)_2_] were sonicated for 10 min in 10 mL of ethanol in an ultrasound bath (room temperature; 80% power). The suspensions were placed directly on a glass surface, and ethanol was allowed to evaporate. After the samples were dry, they were characterized by PXRD and IR.

Excitation and emission studies of [Cu_3_I_5_(bz-ted)_2_], [Cu_4_I_6_(pr-ted)_2_], and [Cu_3_I_5_(bz-ted)_2_]@3D0.24% composites.

In total, 1 mL of an 11 mg/mL suspension of [Cu_3_I_5_(bz-ted)_2_] in ethanol was centrifuged for 10 min at 15,000 rpm. The supernatant was removed with a Pasteur pipette, and the remaining solid was dried in a desiccator for 72 h. Once dry, the excitation and emission spectra were recorded at room temperature. The luminescence measurements were performed using the following excitation and emission wavelengths: λex = 335 nm and λem = 555 nm for [Cu_3_I_5_(bz-ted)_2_]; λex = 326 nm and λem = 520 nm for [Cu_3_I_5_(bz-ted)_2_] @3D_0_._24_%; and λex = 380 nm and λem = 530 nm for [Cu_4_I_6_(pr-ted)_2_].

3D printer processing: [Cu_3_I_5_(bz-ted)_2_]@3D0.24% and [Cu_4_I_6_(pr-ted)_2_]@3D0.1% composites.

Suspensions of [Cu_3_I_5_(bz-ted)_2_] particles in ethanol were prepared at a concentration of 11 mg/mL by vortex mixing for 2 min, followed by sonication in an ultrasonic bath for 3 min at 22 °C and 100% power. In total, 1 mL of this suspension was then mixed with 4 mL of the photopolymer resin to obtain a composite containing 0.24 wt% of [Cu_3_I_5_(bz-ted)_2_]. The resulting mixture was 3D-printed through layer-by-layer UV-induced photopolymerization. The printed geometry consisted of 2.8 × 2.8 cm squares with a thickness of 0.5 mm.

The [Cu_4_I_6_(pr-ted)_2_]@3D0.1% composite was also fabricated by 3D printing, following the procedure previously reported [27].

[Cu_3_I_5_(bz-ted)_2_]@PLA3.6%, and [Cu_4_I_6_(pr-ted)_2_]@PLA3.6% composite films.

Two solutions of 80 mg of polylactic acid (PLA) were prepared, each dissolved in 2 mL of acetonitrile under magnetic stirring (800 rpm) for 1 h at 60 °C. Then, 3 mg of [Cu_3_I_5_(bz-ted)_2_] and 3 mg of [Cu_4_I_6_(pr-ted)_2_] were added to the respective solutions under magnetic stirring (800 rpm) at room temperature for 10 min and subsequently sonicated in a sonication bath (80% power; room temperature) for 10 min to obtain stable suspensions. The resulting suspensions were deposited by drop-casting onto glass slides and left to dry for 1 h until the solvent had completely evaporated.

Water stability of [Cu_3_I_5_(bz-ted)_2_], Cu_4_I_6_(pr-ted)_2_], [Cu_3_I_5_(bz-ted)_2_]@3D0.24%, and [Cu_4_I_6_(pr-ted)_2_]@3D0.1% over time.

The temporal stability of [Cu_3_I_5_(bz-ted)_2_] was evaluated using ^1^H-NMR spectroscopy in D_2_O. A suspension was prepared by dispersing 5 mg of [Cu_3_I_5_(bz-ted)_2_] in 0.6 mL of D_2_O at room temperature. The ^1^H-NMR spectrum of the suspension was first recorded at t = 0 h, and a second spectrum was acquired after 2 h under identical conditions. Additionally, the ^1^H-NMR spectrum was recorded for the bz-ted ligand in D_2_O for comparison with the spectra under study.

The stability of [Cu_4_I_6_(pr-ted)_2_] over time has been previously reported [27] for exposure times from t_1_ = 0 to t_2_ = 7 days.

The stability of the [Cu_3_I_5_(bz-ted)_2_]@3D0.24% composite in D_2_O over time was evaluated using ^1^H NMR spectroscopy. A piece of the composite was immersed in D_2_O, and the ^1^H NMR spectrum was recorded immediately (t = 0) and again after 2 h of immersion at room temperature. The spectra were compared to detect potential degradation.

Water stability of [Cu_3_I_5_(bz-ted)_2_], [Cu_4_I_6_(pr-ted)_2_], [Cu_3_I_5_(bz-ted)_2_]@3D0.24%, and [Cu_4_I_6_(pr-ted)_2_]@3D0.1% across a wide pH range.

The stability of [Cu_3_I_5_(bz-ted)_2_] was studied at different pH values from pH = 2 to pH = 8, which is the relevant range for aqueous sensing applications [27]. Two suspensions of [Cu_3_I_5_(bz-ted)_2_] were prepared in deionized water, and both initially appeared to be white. The first suspension, with an initial pH = 6.75, was acidified to pH = 2.45 by adding small aliquots of 0.1 M HCl. The second suspension, initially at pH = 6.20, was adjusted to pH = 8.21 using drops of 0.01 M NaOH. Subsequently, the solids were separated from the supernatants by centrifugation and analyzed using PXRD.

The stability of [Cu_4_I_6_(pr-ted)_2_] has been previously reported over a pH range of 4–9 [27].

Detection of metal ions and antibiotics with [Cu_3_I_5_(bz-ted)_2_]@3D0.24% and [Cu_4_I_6_(pr-ted)_2_]@3D0.1%.

Selectivity studies

The selectivity of [Cu_3_I_5_(bz-ted)_2_] and [Cu_4_I_6_(pr-ted)_2_] over different metal ions was qualitatively investigated using their corresponding portable composite devices, [Cu_3_I_5_(bz-ted)_2_]@3D0.24% and [Cu_4_I_6_(pr-ted)_2_]@3D0.1%. The ability of these materials to detect various metal ions and, in the case of [Cu_3_I_5_(bz-ted)_2_], select antibiotics, was evaluated under UV light irradiation (λ_exc_ = 365 nm). Aqueous solutions (0.1 M) of Al^3+^, Mg^2+^, Cu^2+^, Ca^2+^, Na^+^, Fe^3+^, Cr^3+^, and Pb^2+^ were prepared. These were obtained by dissolving the corresponding metal nitrates—Al(NO_3_)_3_, Mg(NO_3_)_2_, Cu(NO_3_)_2_, Ca(NO_3_)_2_, NaNO_3_, Fe(NO_3_)_3_, Cr(NO_3_)_3_, and Pb(NO_3_)_2_—in deionized water. Each composite device was immersed in a metal-ion solution and exposed to UV light (λ_exc_ = 365 nm) to observe possible luminescence quenching, allowing a qualitative assessment of each device’s selectivity to take place for specific analytes. All selectivity tests were performed in both deionized water and river water (Manzanares River, Madrid Community, Spain).

Additionally, the sensing capability of [Cu_3_I_5_(bz-ted)_2_]@3D_0.24_% toward antibiotics was investigated. Three representative and commonly used pharmaceuticals from different families were selected: sulfamethazine (SMZs, sulfonamides), chloramphenicol (CAPs, chloramphenicols), and tetracycline (TCs, tetracyclines). The composite device was immersed in 517.5 µM river water solutions of each antibiotic for 30 s under UV light (λ_exc_ = 365 nm).

Sensitivity studies of [Cu_3_I_5_(bz-ted)_2_] and [Cu_4_I_6_(pr-ted)_2_] for Fe (III) ion detection

Once the ability of [Cu_3_I_5_(bz-ted)_2_]@3D0.24% and [Cu_4_I_6_(pr-ted)_2_]@3D0.1% to qualitatively detect specific analytes was confirmed, quantitative studies were carried out using suspensions of submicrometric particles to determine the limit of detection (LOD) for each of them.

Suspensions containing 5 mg of [Cu_3_I_5_(bz-ted)_2_] and Cu_4_I_6_(pr-ted)_2_] particles, respectively, in 5 mL of deionized water were prepared by bath sonication for 10 min at 80% power. In total, 3 mL of these suspensions was transferred to a quartz cuvette and placed in a spectrofluorometer. The emission spectrum was recorded at an excitation wavelength of λex = 335 nm. Then, 50 µL aliquots of a 1 mM Fe(NO_3_)_3_ aqueous solution were successively added to the cuvette, and the emission spectrum was recorded after each addition. Throughout the experiment, the suspension was continuously stirred at 800 rpm. This study was also carried out in river water.

Sensitivity studies of [Cu_3_I_5_(bz-ted)_2_] for detection of tetracycline (TC)

In total, 517.5 µM of a TC solution was prepared by dissolving 2.3 mg of TC in 10 mL of deionized water using a vortex mixer. Separately, 5.4 mg of [Cu_3_I_5_(bz-ted)_2_] was dispersed in 5 mL of deionized water using sonication in an ultrasonic bath at 80% power for 10 min, yielding a homogeneous white suspension. The emission spectrum of [Cu_3_I_5_(bz-ted)_2_] was recorded at an excitation wavelength of 335 nm. Subsequently, emission spectra were acquired after successive additions of 50 µL aliquots of the 517.5 µM TC solution to the aqueous suspension. The luminescence intensity was monitored as a function of antibiotic concentration to evaluate the material’s sensing performance and determine its limit of detection (LOD).

Limit of detection (LOD)

Results from sensitivity studies were used to compute the LOD. LOD values were calculated according to the IUPAC criterion by analyzing the linear correlation between the relative emission intensity (I_0_/I) and analyte concentration, using the Stern–Volmer (S–V) equation [51].

Interference studies of [Cu_3_I_5_(bz-ted)_2_] and [Cu_4_I_6_(pr-ted)_2_] in the presence of metal ions and antibiotics

A 1 mg/mL suspension of the corresponding compound in deionized water was prepared. In total, 3 mL of each suspension was placed into the quartz cuvette and placed onto the spectrofluorometer under magnetic stirring (room temperature; 800 rpm). The emissions of the corresponding suspensions were measured $\left(\lambda_{exc}=335\;nm\;and\;\lambda_{em}=555\;nm\right)$ for [Cu_3_I_5_(bz-ted)_2_] and ($\lambda_{exc}=380\;nm\;and\;\lambda_{em}=530\;nm)$ for [Cu_4_I_6_(pr-ted)_2_]. Later, the interferent and the analyte were added to the corresponding suspension. The same moles of interferent and analyte were added in each case.

Quantitative interference experiments were performed using aqueous 0.01 M nitrate solutions of Al^3+^, Mg^2+^, Cu^2+^, Ca^2+^, Na^+^, Cr^3+^, and Pb^2+^. In each experiment, a 3 mL aqueous suspension of the respective compound was placed in a quartz cuvette, and the emission spectrum was first recorded. Then, 200 µL of each metal nitrate solution was added separately to the suspension, and a new emission spectrum was collected after each addition.

After this measurement, 30 µL of Al^3+^ was added, and a new emission measurement was taken. Then, the same Fe^3+^ mols were added to assess whether the presence of Al^3+^ affects the detection of Fe^3+^. This procedure was repeated for each metal interferent. The results of the interference studies can be found in the Supplementary Materials.

Interference studies in the presence of antibiotics for [Cu_3_I_5_(bz-ted)_2_].

In the case of antibiotics, a similar procedure was followed. A 10^−4^ M aqueous solution of TC and 10^−3^ M aqueous solutions of sulfonamide and chloramphenicol were prepared. After recording the emission spectrum of the corresponding compound solution, 30 µL of sulfonamide was added, and a new emission spectrum was recorded. Once the interferent was present in the solution, 300 µL of TC was added to the cuvette, and a new emission spectrum was recorded. The same process was followed for the chloramphenicol interferent.

Experiments to elucidate the detection mechanisms of [Cu_3_I_5_(bz-ted)_2_] and [Cu_4_I_6_(pr-ted)_2_].

The luminescent compounds and the analyte were mixed in deionized water and stirred for 5 min. Afterwards, the solids were separated from the solutions by centrifugation, and the supernatant was removed. The recovered powder was washed with deionized water to remove traces of the analyte and dried under vacuum. The X-ray diffractogram of the recovered material was then recorded and compared with that of the pristine compound. The inner filter effect (IFE) can be experimentally verified by comparing the UV–Vis absorption spectra of the luminescent compound, the analyte, and their mixture.

Studies of the recyclability of [Cu_4_I_6_(pr-ted)_2_])_2_]@3D0.24%, and [Cu_3_I_5_(bz-ted)_2_]@3D0.24% composites.

All recyclability experiments were carried out following a previously reported procedure [38]. For [Cu_4_I_6_(pr-ted)_2_] submicrometric particles, an aqueous suspension was prepared, and 10 mL of an aqueous Fe(III) solution (400 μM) was added. After allowing the mixture to interact for 5 min under magnetic stirring (800 rpm; room temperature), the suspension was centrifuged at 5000 rpm for 10 min. The supernatant was discarded, and the solid was washed twice with 5 mL of deionized water to remove any residual Fe(III). The recovered solid was then assessed using PXRD and compared with the pristine compound.

Since some powder is lost during each cycle, the mass available after each cycle should be limited to ensure the recyclability studies are more easily evaluated with 3D composite devices.

For both composites, recyclability was evaluated under exposure to both tetracycline (TC) and Fe(III) over 20 consecutive cycles each. Each cycle involved immersing the composites for 10 s in a 0.01 M solution of Fe^3+^ or a 517.5 μM solution of TC, depending on the analyte.

## 4. Conclusions

This study demonstrates that Cu(I)–iodide coordination compounds [Cu_3_I_5_(bz-ted)_2_] and [Cu_4_I_6_(pr-ted)_2_] represent an exceptional class of photoluminescent sensors for environmental water monitoring. The most significant finding is the exceptional sensitivity achieved by [Cu_3_I_5_(bz-ted)_2_], which exhibits a limit of detection (LOD) of 0.038 nM for the detection of tetracycline in river water—substantially outperforming most reported luminescent sensors. Both compounds display nanomolar detection limits for Fe(III) ions (1.33–1.58 nM), demonstrating competitive performance compared to state-of-the-art coordination polymers and metal–organic frameworks. These ultralow detection thresholds, well below environmental and regulatory limits, establish these materials as viable alternatives to conventional analytical instrumentation.

The controlled reduction in particle size through ultrasonic treatment and ball milling proved highly effective for generating stable nanometric suspensions that preserve the optical and structural properties of the parent compounds. The subsequent incorporation of these materials into polymeric matrices via 3D printing and drop-casting yields mechanically robust, low-cost composite devices that maintain full sensing functionality in aqueous environments. Although bare particles show time-dependent degradation in water, polymer encapsulation effectively stabilizes the luminescent compounds, enabling extended operation in real environmental samples, including river water. This practical solution overcomes a critical limitation of many Cu(I) halide systems and renders these sensors suitable for field deployment.

Photoinduced electron transfer (PET) was identified as the dominant quenching mechanism through control experiments, demonstrating the preservation of a crystalline structure and recovery from reversible luminescence. The absence of chemical reactions between the sensor material and analytes, combined with the reversibility of the sensing response, confirms that the quenching process involves no permanent modification of the luminophore. This fundamental finding is crucial, as it enables the reusability of these sensors without loss of performance—a property rarely achieved in luminescent sensing platforms. The mechanism explored in this work enhances our understanding of how cluster nuclearity and ligand environment govern excited-state reactivity and analyte selectivity in Cu(I) halide systems.

The exceptional recyclability demonstrated over 20 consecutive sensing cycles without degradation of emission intensity or structural integrity directly addresses sustainability concerns and dramatically reduces the operational cost of these sensors. The use of earth-abundant copper, readily available organic ligands, and facile processing routes in commercial 3D printing and simple drop-casting methods results in low-cost, portable devices accessible to resource-limited settings and field-based environmental surveys. These advantages—combined with the ultralow detection limits and operation in real-water matrices—position Cu(I)–iodide/polymer composites as promising candidates for practical implementation in drinking water quality monitoring and wastewater treatment applications.

## Figures and Tables

**Table 1:** Detection limits of tetracycline (TC) and iron(III) in water using different compounds and methodologies.

Compound/Methodology	LOD (nM) TC/Fe(III)	Ref.
HPLC	1.1/14.3	[41]
LnMOF	2.27/147	[38,42]
Silver nanoparticles	0.43/3.6	[43]
Gold nanoparticles	0.2/11.3	[44]
[Zn(QDA)]·0.5H_2_O·0.7DMF ^1^	33/23	[45]
[Zn(L_1_)hfdba] ^2^	/45	[46]
[Cu_3_I_5_(bz-ted)_2_]	0.038/1.33	This reference
[Cu_4_I_6_(pr-ted)_2_]	/1.58	This reference
[Cu_4_I_6_(pr-ted)_2_]	1.18/	[27]

^1^ H_2_QDA: quinoline-2,6-dicarboxylic acid, DMF = N,N-dimethylformamide. ^2^ H_2_hfdba: 2,2-bis(4-carboxyphenyl)hexafluoropropane.

## Data Availability

The data supporting this article are provided in the Supplementary Materials.

## Supplementary Materials

The following supporting information can be downloaded at https://www.mdpi.com/article/10.3390/molecules31091384/s1. Figure S1: ^1^H-RMN of bz-ted in D_2_O at room temperature; Figure S2: ^1^H-RMN of pr-ted in D_2_O at room temperature; Figure S3: FT-IR spectra of bz-ted (orange) and [Cu_3_I_5_(bz-ted)_2_] (blue); Figure S4: PXRD of the obtained [Cu_3_I_5_(bz-ted)_2_] (blue) and the theoretical one by single-crystal X-ray diffraction (SXRD) (yellow); Figure S5: FT-IR spectra of pr-ted (black) and [Cu_4_I_6_(pr-ted)_2_] (red); Figure S6: PXRD of the obtained [Cu_4_I_6_(pr-ted)_2_] (red) and the theoretical one by SXRD (black); Figure S7: SEM images of [Cu_3_I_5_(bz-ted)_2_]_with different magnifications: (a) ×1 kX (b) ×50 kX; Figure S8: SEM images of [Cu_4_I_6_(pr-ted)_2_]_with different magnifications: (a) ×10 kX (b) ×25 kX; Figure S9: FT-IR spectra of [Cu_3_I_5_(bz-ted)_2_] (black) and [Cu_3_I_5_(bz-ted)_2_] after 10 min of sonication (80% power; 25 °C) in ethanol (red); Figure S10: FT-IR spectra of [Cu_4_I_6_(pr-ted)_2_](black) and [Cu_4_I_6_(pr-ted)_2_] after 10 min of sonication (80% power; 25 °C) in ethanol (red); Figure S11: PXRD of [Cu_3_I_5_(bz-ted)_2_] (black) and [Cu3I5(bz-ted)2] after 10 min of sonication (80% power; 25 °C) in ethanol (red); Figure S12: PXRD of [Cu_4_I_6_(pr-ted)_2_] (black) and [Cu_4_I_6_(pr-ted)_2_] after 10 min of sonication (80% power; 25 °C) in ethanol (red); Figure S13: AFM images of [Cu_3_I_5_(bz-ted)_2_] after 10 min of sonication (60% power; 25 °C) in ethanol. Obtained by deposition on SiO_2_; Figure S14: AFM images of [Cu_3_I_5_(bz-ted)_2_] after 10 min of sonication (80% power; 25 °C) in ethanol. Obtained by deposition on SiO_2;_ Figure S15: SEM (a) and AFM (b) images of Cu3I5(bz-ted)2 after 10 min of sonication (80% power; 25 °C, room temperature) in ethanol. Figure S16: FT-IR spectra [Cu_3_I_5_(bz-ted)_2_] obtained by ball milling (black) and [Cu_3_I_5_(bz-ted)_2_] obtained by PVP (red). Figure S17: PXRD patterns of [Cu_3_I_5_(bz-ted)_2_]_ball milling (red) compared with its theoretical diffraction pattern (black); Figure S18: SEM images of [Cu_3_I_5_(bz-ted)_2_] obtained by ball milling at different magnifications (25 KX and 50 KX); Figure S19: [Cu_3_I_5_(bz-ted)_2_] obtained by ball milling after 10 min of sonication (80% power; 25 °C) in ethanol at different magnifications: (a) 25 KX and (b) 50 KX; Figure S20: Emission of [Cu_4_I_6_(pr-ted)_2_] (green) and [Cu_3_I_5_(bz-ted)_2_] (orange-yellow) under UV light (365 nm); Figure S21: Excitation (red) (λ_em_ = 530 nm), and emission (black) (λ_ex_ = 380 nm) spectra of [Cu_4_I_6_(pr-ted)_2_] suspended in deionized water; Figure S22: Excitation (red) (λ_em_ = 530 nm), and emission (black) (λ_ex_ = 380 nm) spectra of [Cu_3_I_5_(pr-ted)_2_] obtained by ball milling suspended in deionized water. Figure S23: ^1^H-NMR spectra of [Cu_3_I_5_(bz-ted)_2_] in D_2_O at (a) t_0_ = 0 and b) t_1_ = 2 h; Figure S24: PXRD pattern of [Cu_3_I_5_(bz-ted)_2_] (blue), [Cu_3_I_5_(bz-ted)_2_] pH = 2 (orange), and [Cu_3_I_5_(bz-ted)_2_] pH = 8 (green); Figure S25: IR spectra of the [Cu_3_I_5_(bz-ted)_2_]@3D0.91%, (black) and Standard Photopolymer Translucid Resin (red); Figure S26: IR spectra of [Cu_4_I_6_(pr-ted)_2_]@3D0.1% (black) and Standard Photopolymer Translucid Resin; Figure S27: PXRD of the obtained [Cu_3_I_5_(bz-ted)_2_] (blue), [Cu_3_I_5_(bz-ted)_2_]@3D0.24% (green) and Standard Photopolymer Translucid Resin (purple); Figure S28: XRD theoretical pattern of [Cu_4_I_6_(pr-ted)_2_] (black), PXRD of [Cu_4_I_6_(pr-ted)_2_]@3D0.24% (red) and Standard Photopolymer Translucid Resin (blue); Figure S29: [Cu_3_I_5_(bz-ted)_2_]@3D0.24% excitation spectrum (orange) (λ_em_ = 520 nm) and [Cu_3_I_5_(bz-ted)_2_]@3D0.24% emission spectrum (blue) (λ_ex_ = 326 nm) in solid state; Figure S30: FT-IR spectra of [Cu_3_I_5_(bz-ted)_2_] (black), [Cu_3_I_5_(bz-ted)2]@PLA3,6% (red) and PLA_acetonitrile (blue); Figure S31: FT-IR spectra of [Cu_4_I_6_(pr-ted)_2_] (black), [Cu_4_I_6_(pr-ted)2]@PLA3.6% (red) and PLA_acetonitrile (blue); Figure S32: PXRD of [Cu_4_I_6_(pr-ted)_2_] (blue), [Cu_4_I_6_(pr-ted)_2_]@PLA3.6% (black) and PLA in acetonitrile film (red); Figure S33: PXRD of [Cu_3_I_5_(bz-ted)_2_] (blue), [Cu_3_I_5_(bz-ted)2]@PLA% (black) and PLA in acetonitrile film (red); Figure S34. SEM-EDX map of [Cu_3_I_5_(bz-ted)_2_] distribution in the PLA film ([Cu_3_I_5_(bz-ted)_2_]@PLA3.4%) (left image) and SEM image of a specific region of the [Cu_3_I_5_(bz-ted)_2_]@PLA3.4% composite (right image); Figure S35: ^1^H-RMN spectrum of [Cu_3_I_5_(bz-ted)_2_]@3D0.24% in D_2_O at t_0_ = 0 (a), and after 2 h (b); Figures S36 and S37: (a) Emission spectra of an aqueous suspension of submicrometric particles of [Cu_3_I_5_(bz-ted)_2_] with respect to the Fe(III) concentration in deionized and river (S37)water, (b) S-V diagram for the quenching of [Cu_3_I_5_(bz-ted)_2_] in deionized water in the presence of Fe (III) in the concentration range 0–1000 µM: Figures S38 and S39: (a) Emission spectra of an aqueous suspension of submicrometric particles of [Cu_3_I_5_(bz-ted)_2_] with respect to the TC concentration in river and desionized (S39) water, (b) S-V diagram for the quenching of [Cu_3_I_5_(bz-ted)_2_] in river water in the presence of TC in the concentration range 20–70 µM; Figure S40: [Cu_3_I_5_(bz-ted)_2_] before (red) and after (black) interacting with Fe(III); Figure S41: [Cu_3_I_5_(bz-ted)_2_] emission at room temperature (red) and Fe(NO_3_)_3_ absorbance (black); Figure S42: Study of the interference of [Cu_3_I_5_(bz-ted)_2_] with different metal ions for the detection of Fe(III). Pristine emission (yellow), emission after addition of 200 µL of a 0.01 M aqueous solution of the interfering metal ion (dark blue), and emission after addition of 200 µL of a 0.01 M aqueous solution of Fe(III) (grey)*;* Figure S43: Study of interference by antibiotics on the emission of [Cu_3_I_5_(bz-ted)_2_]: pristine emission (dark blue), emission after addition of 30 µL of a 10^−3^ M aqueous solution of the interfering antibiotic (dark grey), and emission after addition of 300 µL of a 10^−4^ M aqueous solution of TC (grey); Figure S44: Study of interference by metal ions on the emission of [Cu_4_I_6_(pr-ted)_2_]: pristine emission (light green), emission after addition of 200 µL of a 0.01 M aqueous solution of the interfering metal ion (light blue), and emission after addition of 200 µL of a 0.01 M aqueous solution of Fe(III) (grey): Figure S45: Study of interference by antibiotics on the emission of [Cu_4_I_6_(pr-ted)_2_]: pristine emission (dark blue), emission after addition of 30 µL of a 10^−3^ M aqueous solution of the interfering antibiotic (dark…), and emission after addition of 300 µL of a 10^-4 M aqueous solution of TC (purple); Figure S46: PXRD patterns of [Cu_3_I_5_(bz-ted)_2_]@3D0.24%, after 20 cycles of Fe (III) detection. Simulated [Cu_3_I_5_(bz-ted)_2_] (blue), [Cu_3_I_5_(bz-ted)_2_]@3D_0_._24_% (purple), and Standard Photopolymer Translucid Resin (green); Figure S47. PXRD patterns of [Cu_3_I_5_(bz-ted)_2_]@3D0.24%, after 20 cycles of Fe (III) detection. Simulated [Cu_3_I_5_(bz-ted)_2_] (blue), [Cu_3_I_5_(bz-ted)_2_]@3D_0_._24_% (purple), and Standard Photopolymer Translucid Resin (green).
