# Supplementary material for: Organic–Inorganic Triethylenediamine Cu(I)-Iodides as Reusable Photoluminescent Sensors for Waterborne Pollutants
Source: Molecules. 2026 Apr 22;31(9):1384. doi: 10.3390/molecules31091384 (PMC13165235; doi:10.3390/molecules31091384)
Supplement: Supplementary file 1 [file molecules-31-01384-s001.zip › molecules-4238867-supplementary.pdf]

Organic- Inorganic Triethylenediamine Cu(I)–Iodides as Reusable Photoluminescent Sensors for Waterborne Pollutants

*Victoria Martín[1], Giulia Bardelli[1], Julián Ávila Durán[1] Pilar Amo Ochoa[1,2]**

[1] *Inorganic Chemistry Department, Faculty of Sciences, Autonomous University of Madrid (UAM), 28049 Madrid, Spain.*

[2]*Institute for Advanced Research in Chemical Sciences (IAdChem). Autonomous University of Madrid (UAM), 28049 Madrid, Spain*

Supporting Information

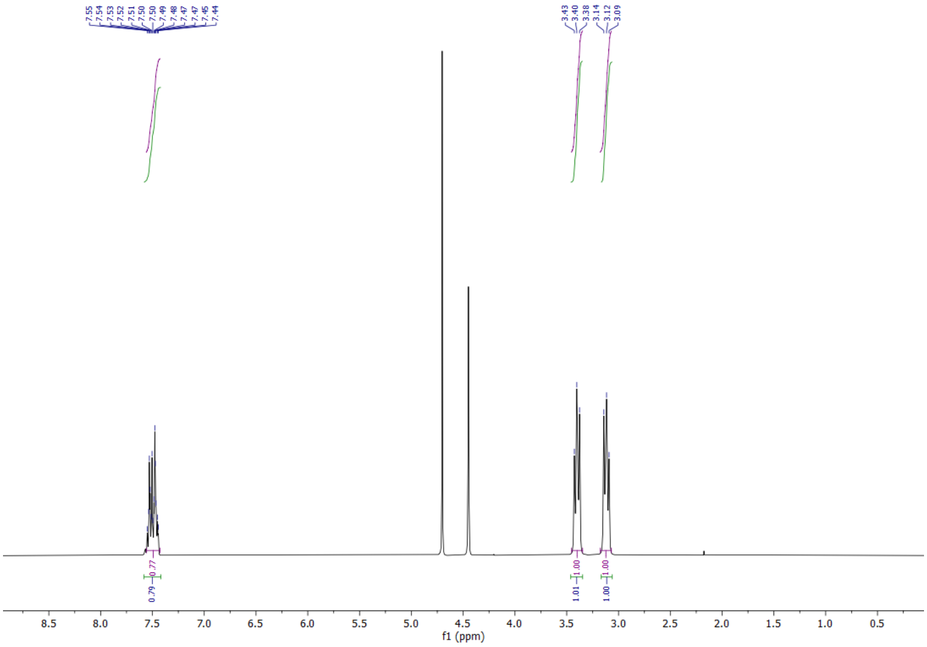

Figure S1: ^{1}H-RMN of bz-ted in D_2O at room temperature.

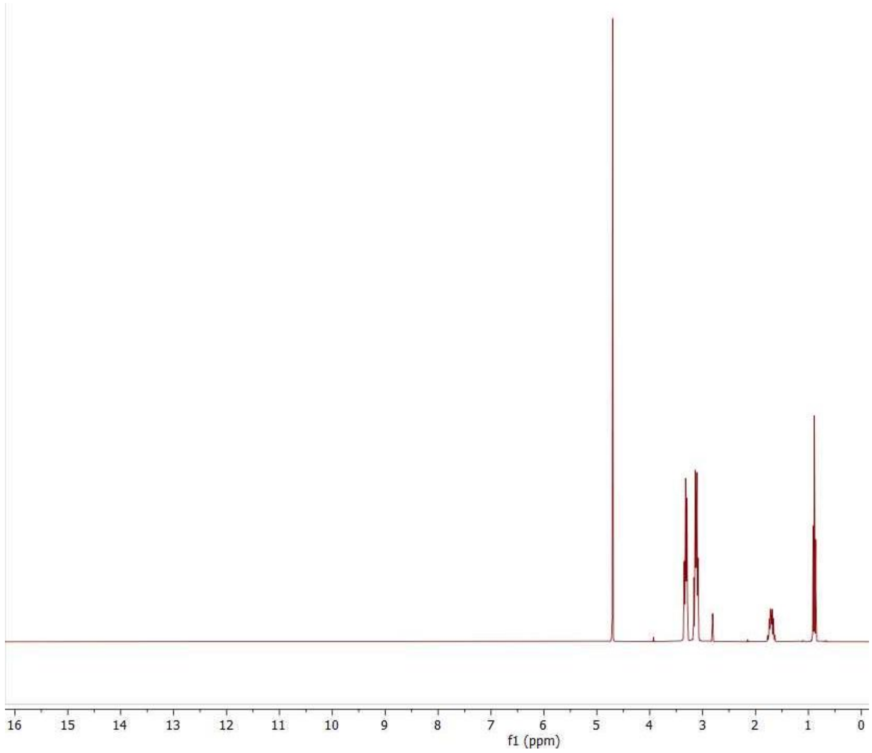

Figure S2: ^{1}H-RMN of pr-ted in D_2O at room temperature.

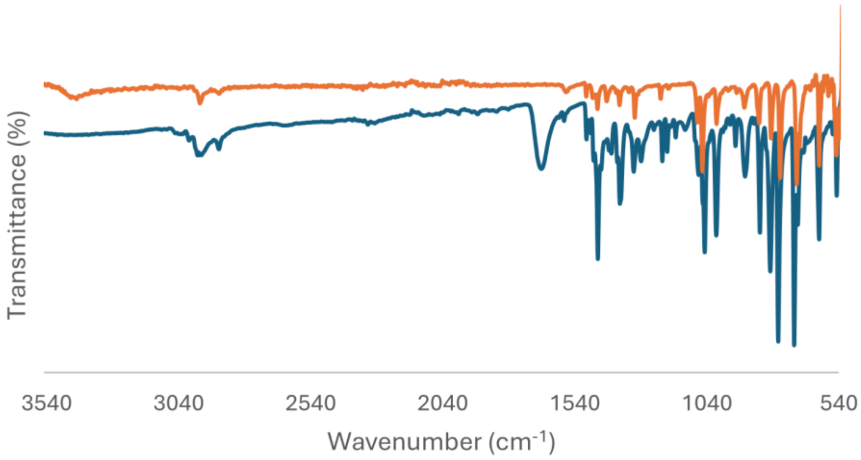

Figure S3: FT-IR spectra of bz-ted (orange) and $[Cu_3I_5(bz\text{-}ted)_2]$ (blue).

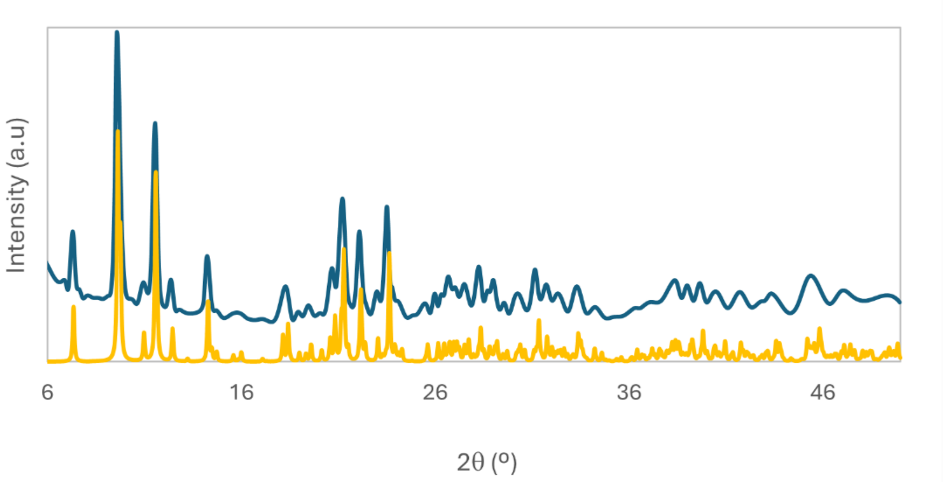

Figure S4: PXRD of the obtained [Cu_3I_5(bz-ted)$_2$](blue) and the theoretical one by single crystal x-ray diffraction (SXRD) (yellow).

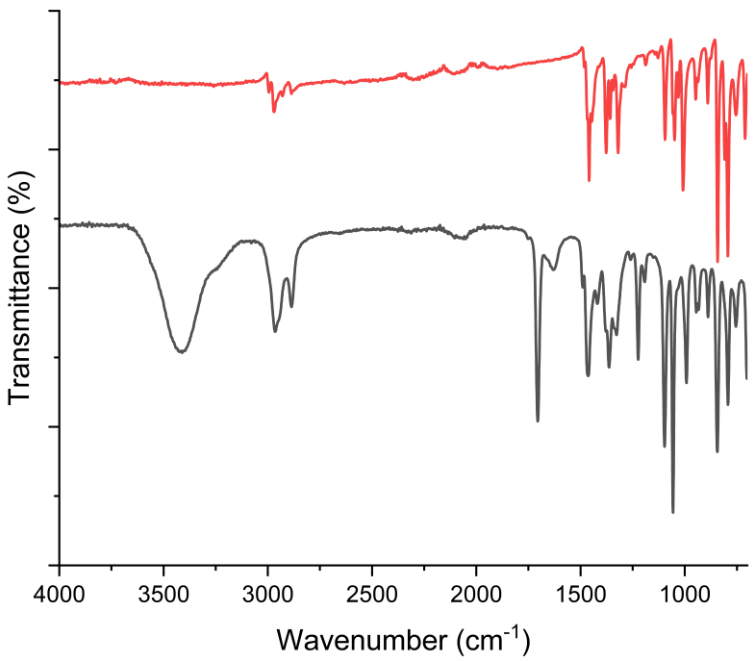

Figure S5: FT-IR spectra of pr-ted (black) and [Cu_4I_6(pr-ted)$_2$] (red).

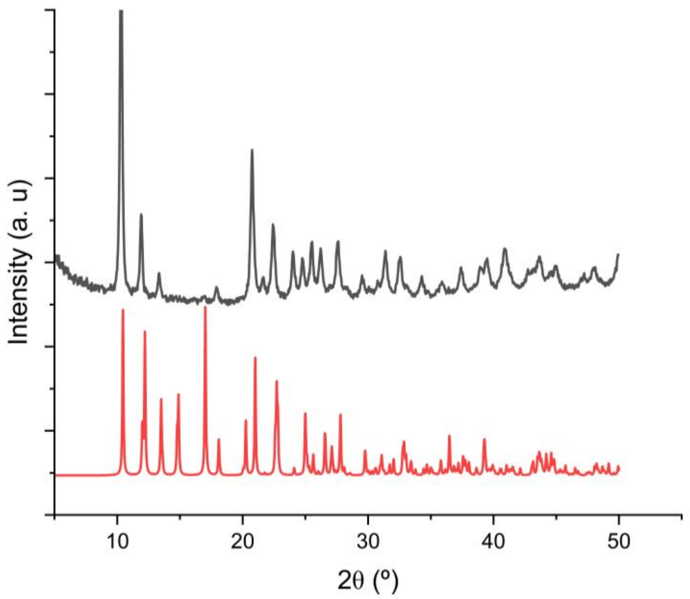

Figure S6: PXRD of the obtained [Cu_4I_6(pr-ted)$_2$] (red) and the theoretical one by SXRD (black).

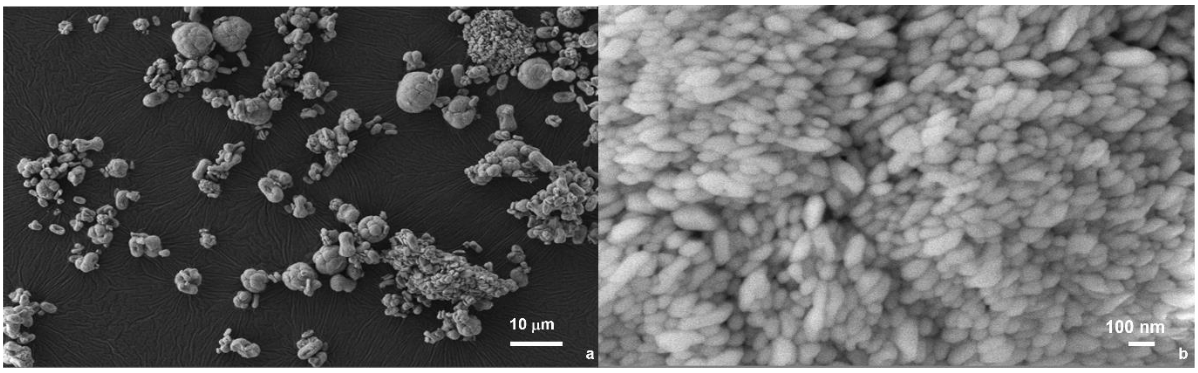

Figure S7: SEM images of [Cu_3I_5(bz-ted)$_2$]_with different magnification a) x 1 kX (b) x 50 kX.

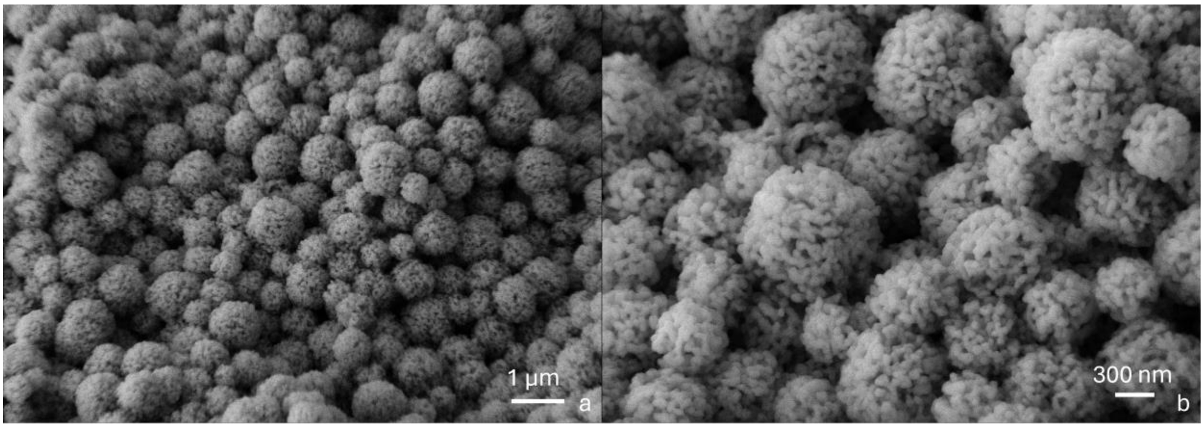

Figure S8: SEM images of [Cu_4I_6(pr-ted)$_2$]_with different magnification a) x 10 kX (b) x 25 kX.

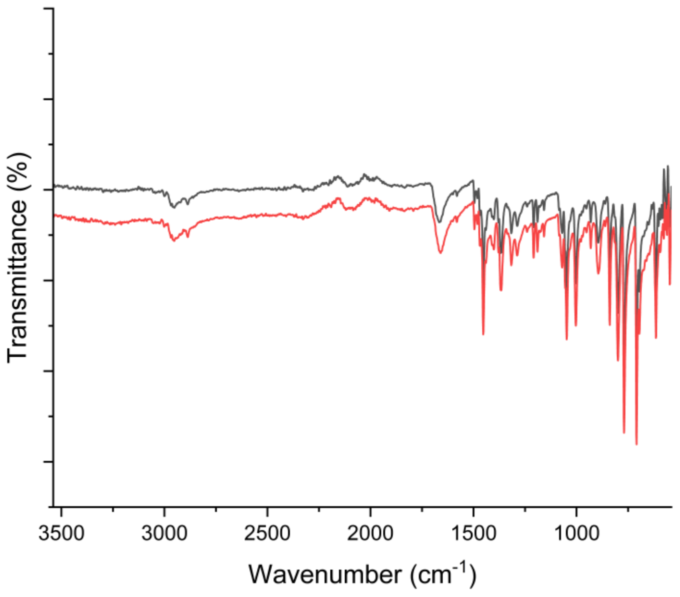

Figure S9: FT-IR spectra of [Cu_3I_5(bz-ted)$_2$] (black) and [Cu_3I_5(bz-ted)$_2$] after 10 mins sonication (80% power, 25 ºC) in ethanol (red).

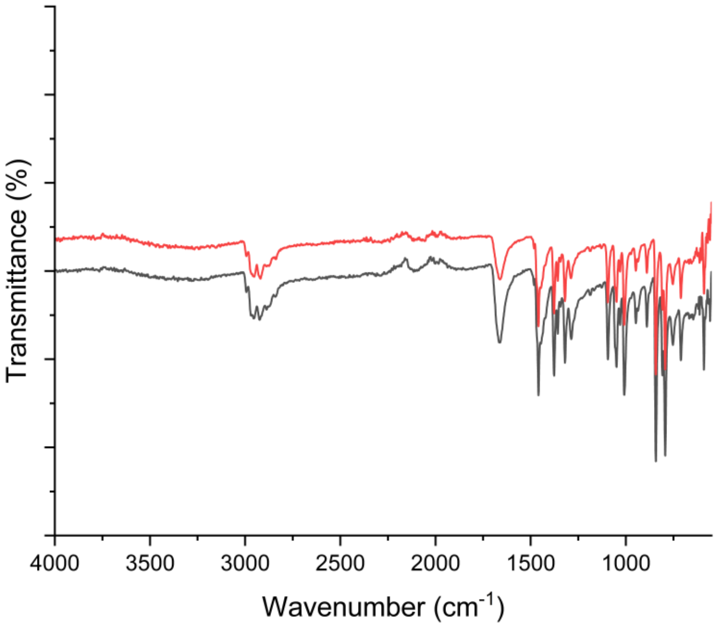

Figure S10: FT-IR spectra of [Cu_4I_6(pr-ted)$_2$](black) and [Cu_4I_6(pr-ted)$_2$] after 10 mins sonication (80% power, 25 ºC) in ethanol (red).

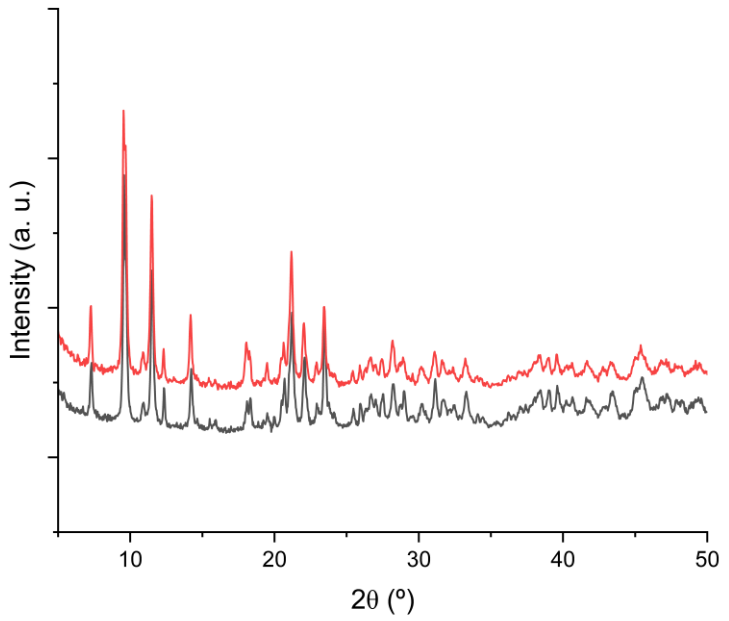

Figure S11: PXRD of $[Cu_3I_5(bz\text{-}ted)_2]$ (black) and $[Cu_3I_5(bz\text{-}ted)_2]$ after 10 minutes sonication (80% power, 25 ºC) in ethanol (red).

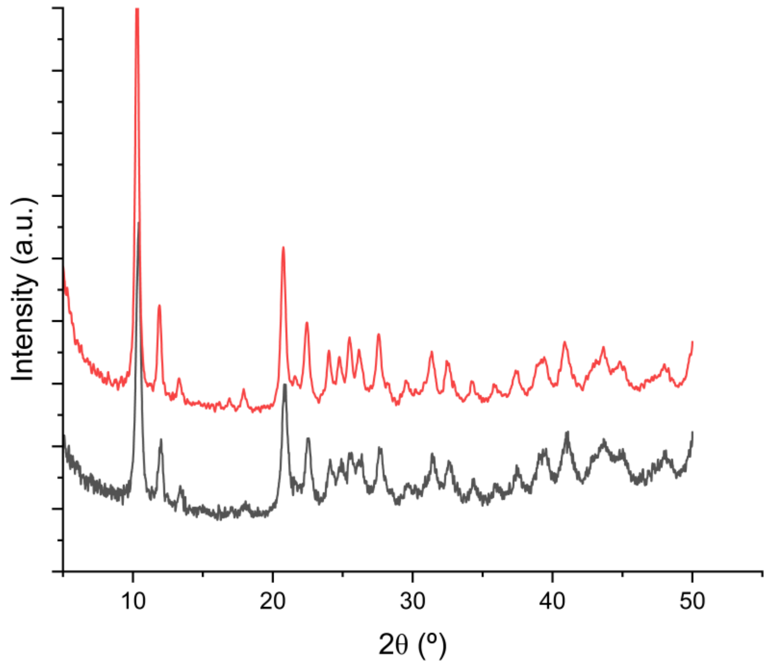

Figure S12: PXRD of $[Cu_4I_6(pr\text{-}ted)_2]$ (black) and $[Cu_4I_6(pr\text{-}ted)_2]$ after 10 minutes sonication (80% power, 25 ºC) in ethanol (red).

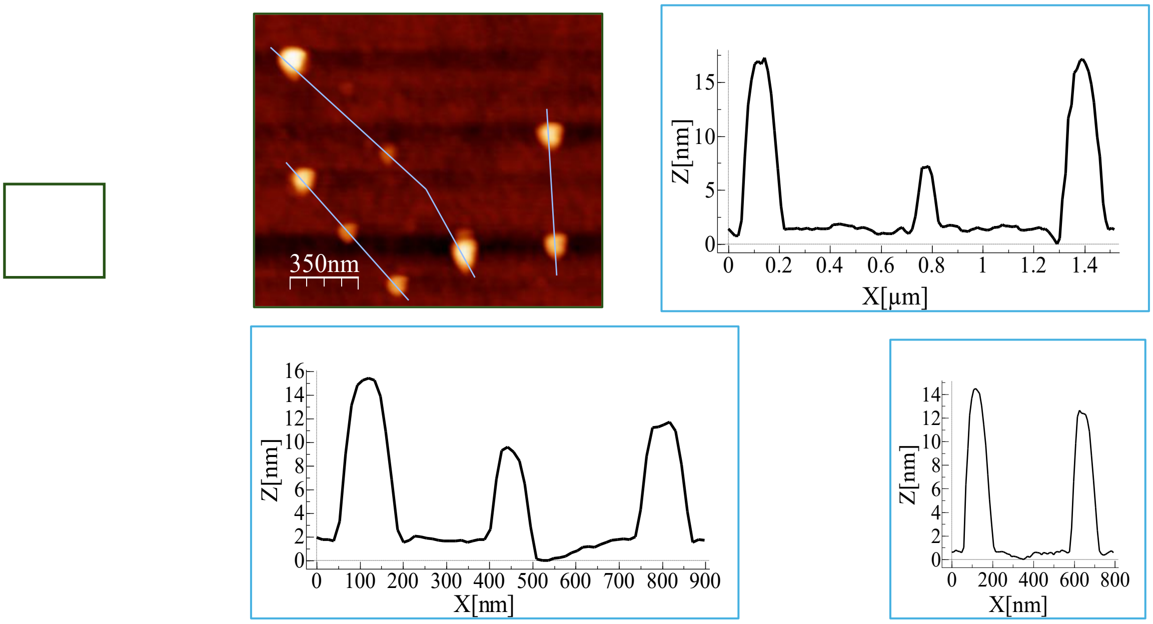

Figure S13. AFM images of [Cu_3I_5(bz-ted)$_2$] after 10 minutes sonication (60% power, 25 ºC) in ethanol. Obtained by deposition on SiO_2.

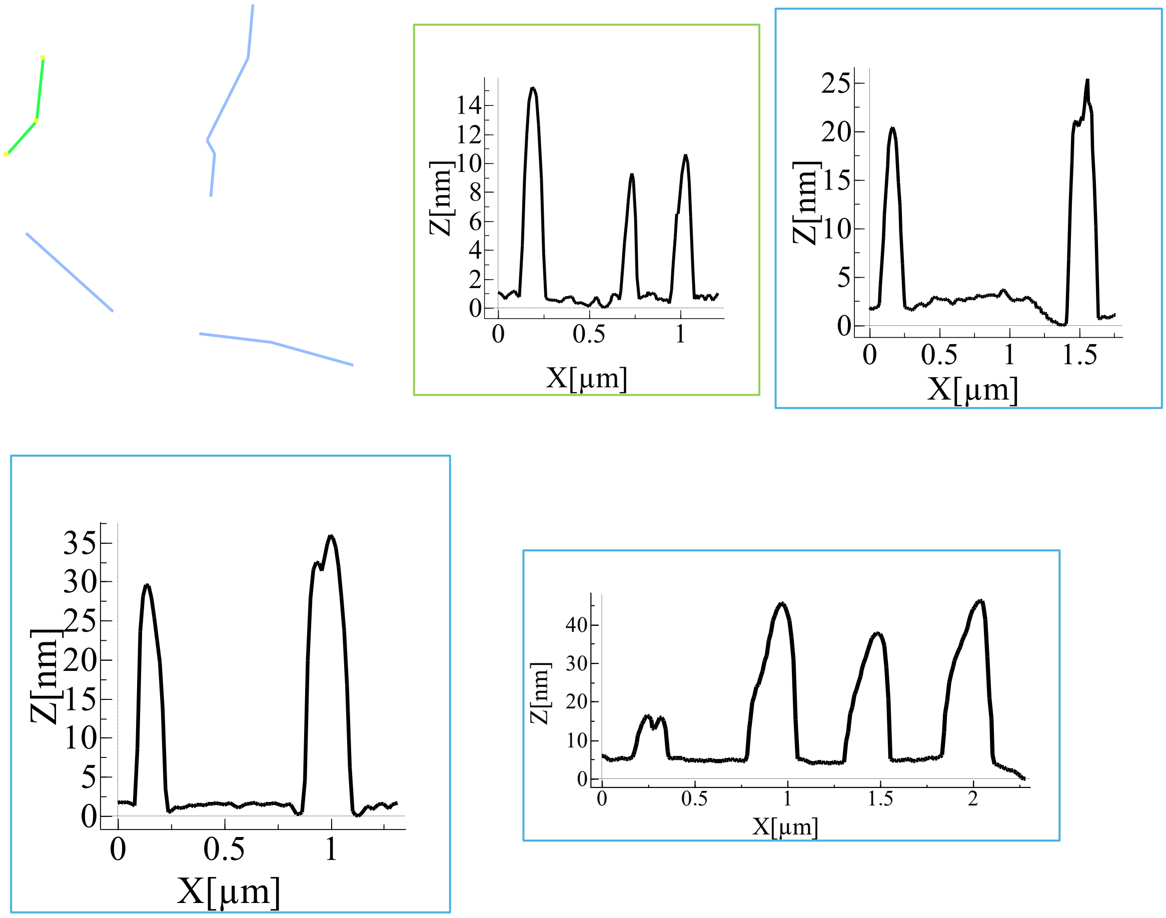

Figure S14. AFM images of [Cu_3I_5(bz-ted)$_2$] after 10 minutes sonication (80% power, 25 ºC) in ethanol. Obtained by deposition on SiO_2

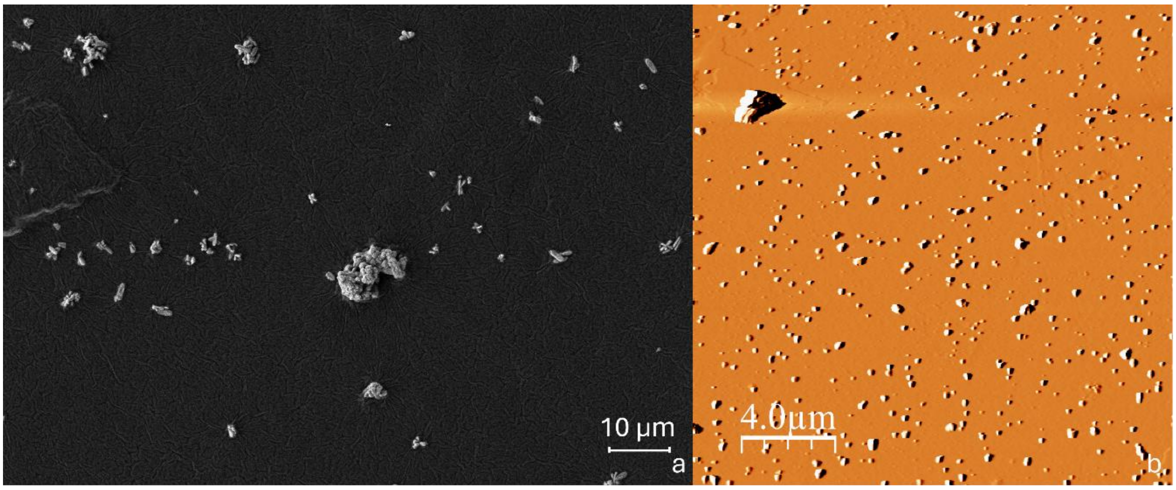

Figure S15. SEM (a) and AFM (b) images of Cu_3I_5(bz-ted)$_2$ after 10 minutes sonication (80% power, 25 ºC, room temperature) in ethanol.

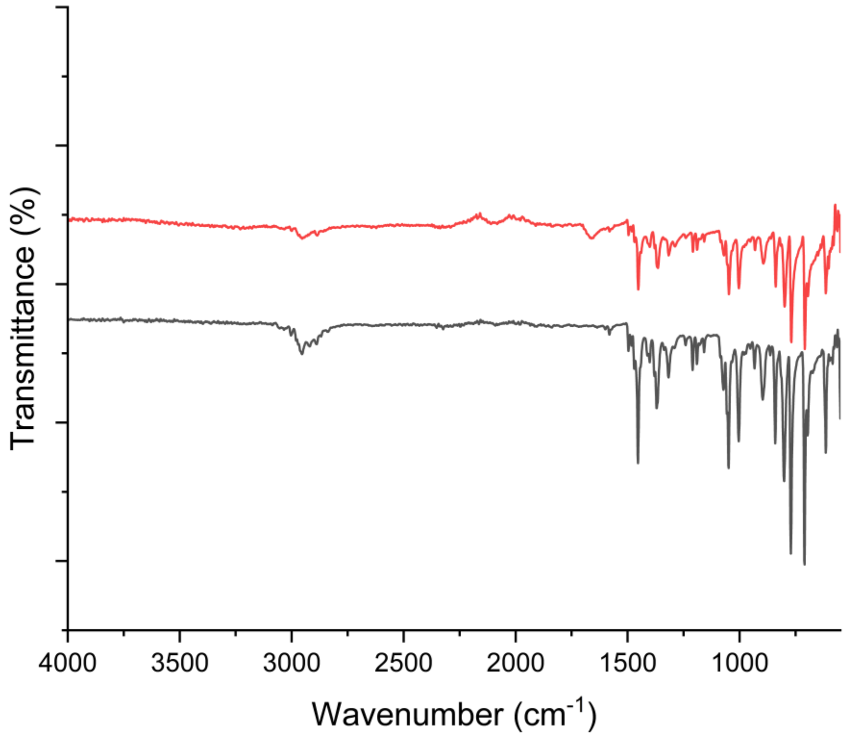

Figure S16. FT-IR spectra [Cu_3I_5(bz-ted)$_2$] obtained by ball milling (black) and [Cu_3I_5(bz-ted)$_2$] obtained by PVP (red).

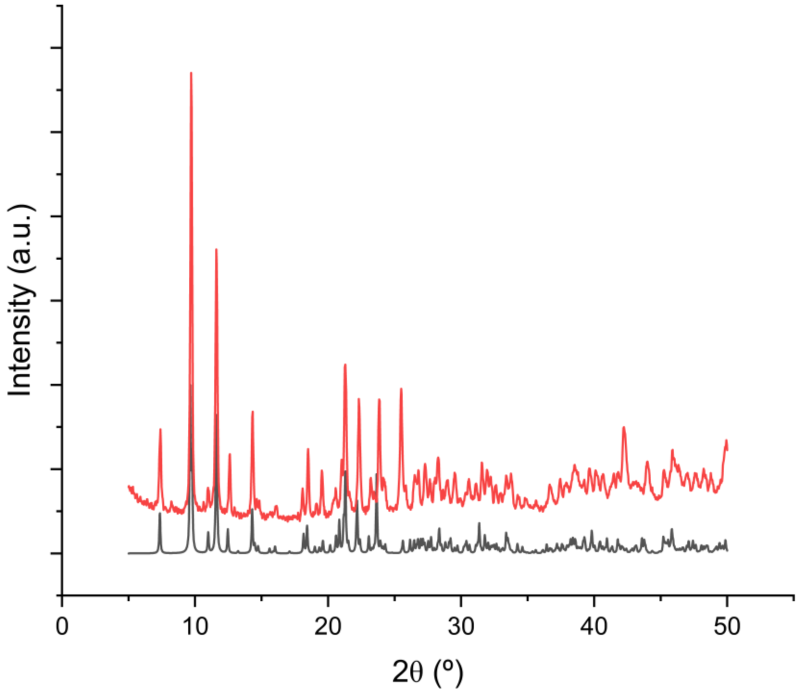

Figure S17: PXRD patterns of $[Cu_3I_5(bz\text{-}ted)_2]$_ball milling (red) compared with its theoretical diffraction pattern (black).

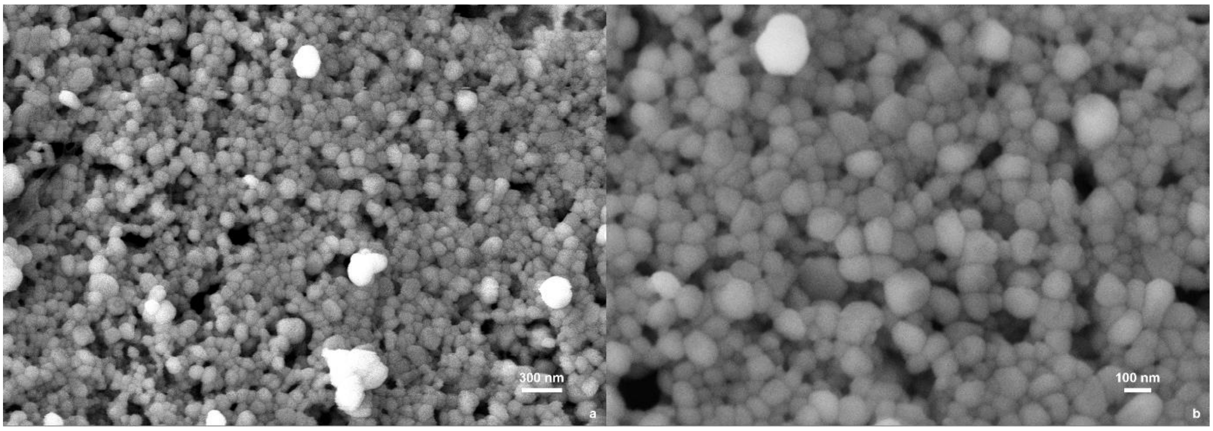

Figure S18: SEM images of $[Cu_3I_5(bz\text{-}ted)_2]$ obtained by ball milling at different magnification (25KX and 50 KX)

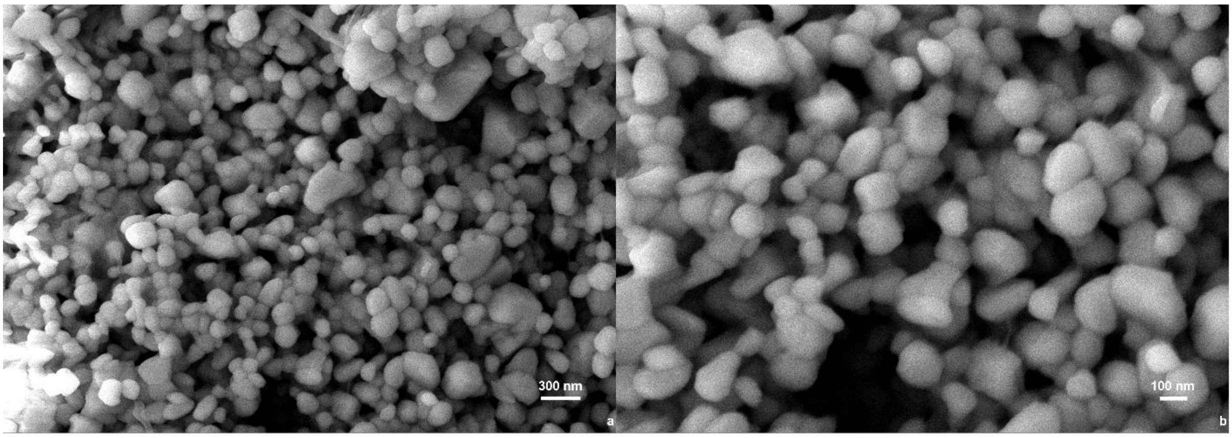

Figure S19. [Cu_3I_5(bz-ted)$_2$] obtained by ball milling after 10 minutes sonication (80% power, 25 ºC) in ethanol at different magnification (a)25KX and (b) 50 KX.

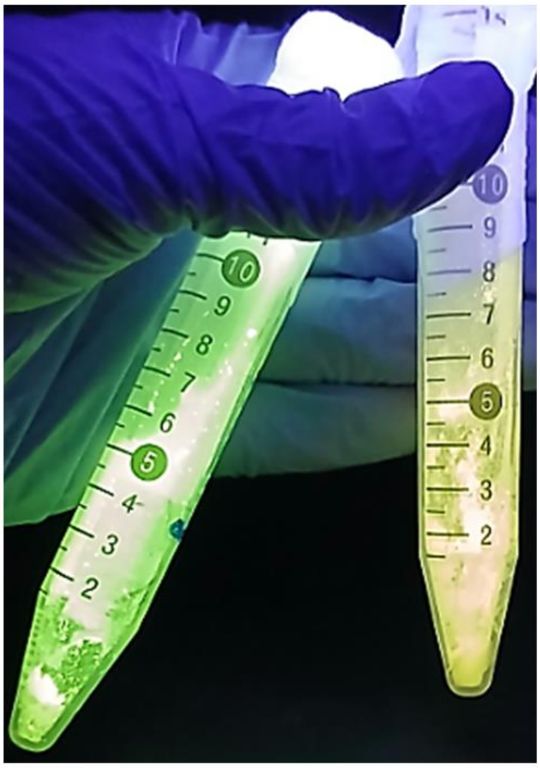

Figure S20: Emission of [Cu_4I_6(pr-ted)$_2$] (green) and [Cu_3I_5(bz-ted)$_2$] (orange-yellow) under UV light (365 nm).

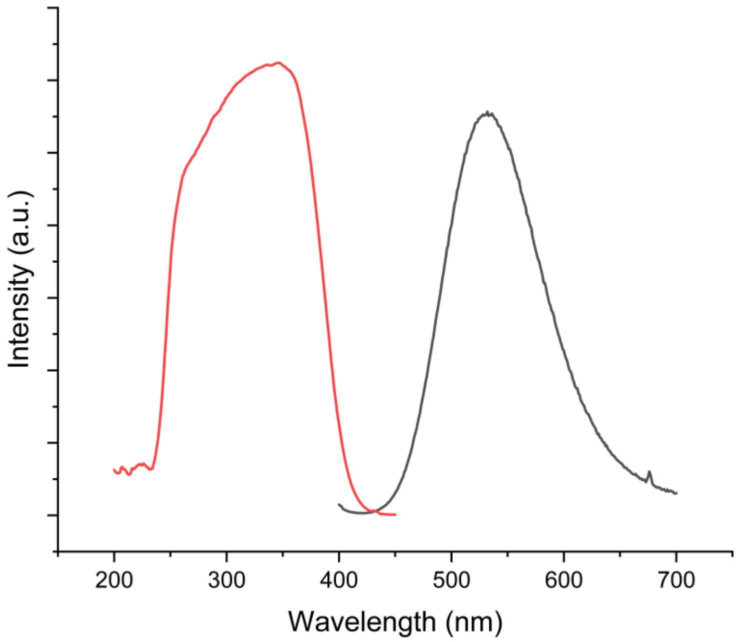

Figure S21: Excitation (red) (λ_{em}= 530 nm), and emission (black) (λ_{ex}= 380 nm) spectra of [Cu_4I_6(pr-ted)$_2$] suspended in deionized water.

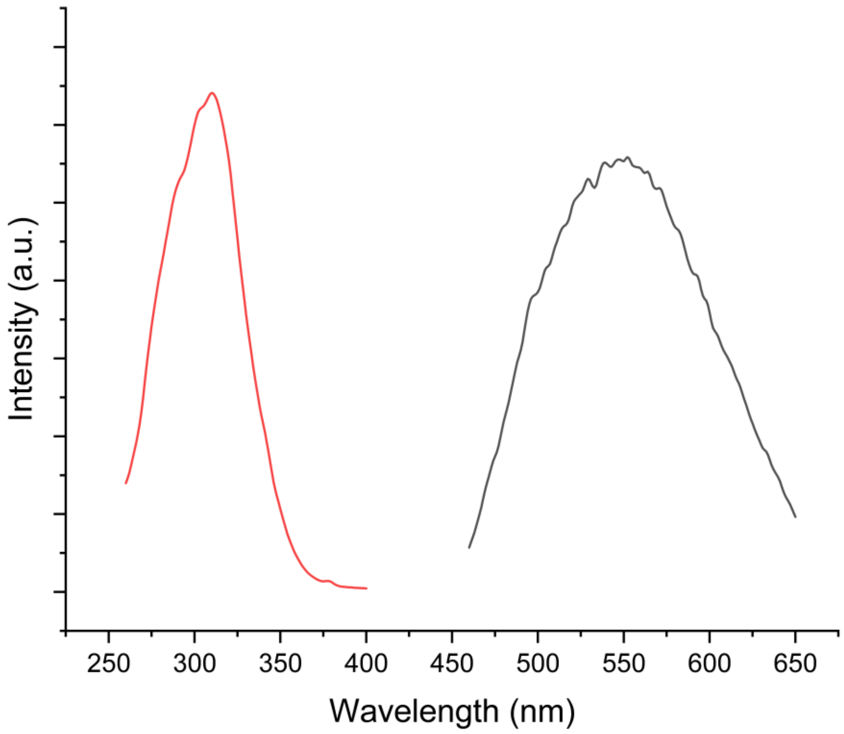

Figure S22: Excitation (red) (λ_{em}= 530 nm), and emission (black) (λ_{ex}= 380 nm) spectra of [Cu_3I_5(pr-ted)$_2$] obtained by ball milling suspended in deionized water.

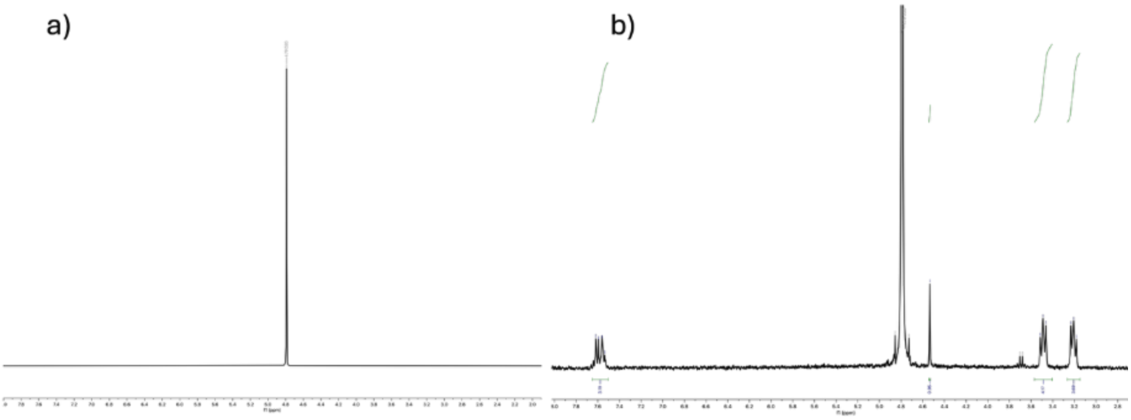

Figure S23: ^{1}H-NMR spectra of $[Cu_3I_5(bz\text{-}ted)_2]$ in D_2O at a) t_0= 0 and b) t_1= 2h

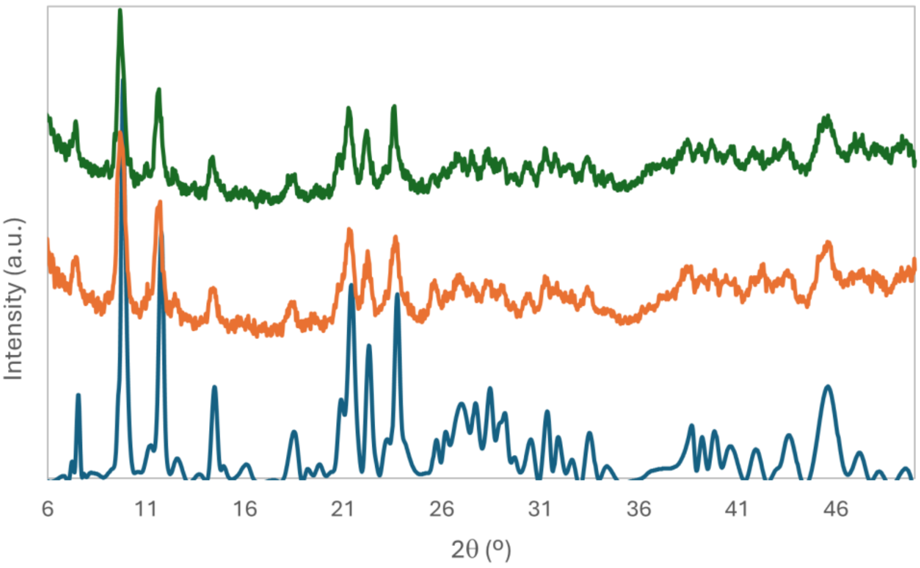

Figure S24. PXRD pattern of $[Cu_3I_5(bz\text{-}ted)_2]$ (blue), $[Cu_3I_5(bz\text{-}ted)_2]$ pH=2 (orange), and $[Cu_3I_5(bz\text{-}ted)_2]$ pH=8 (green).

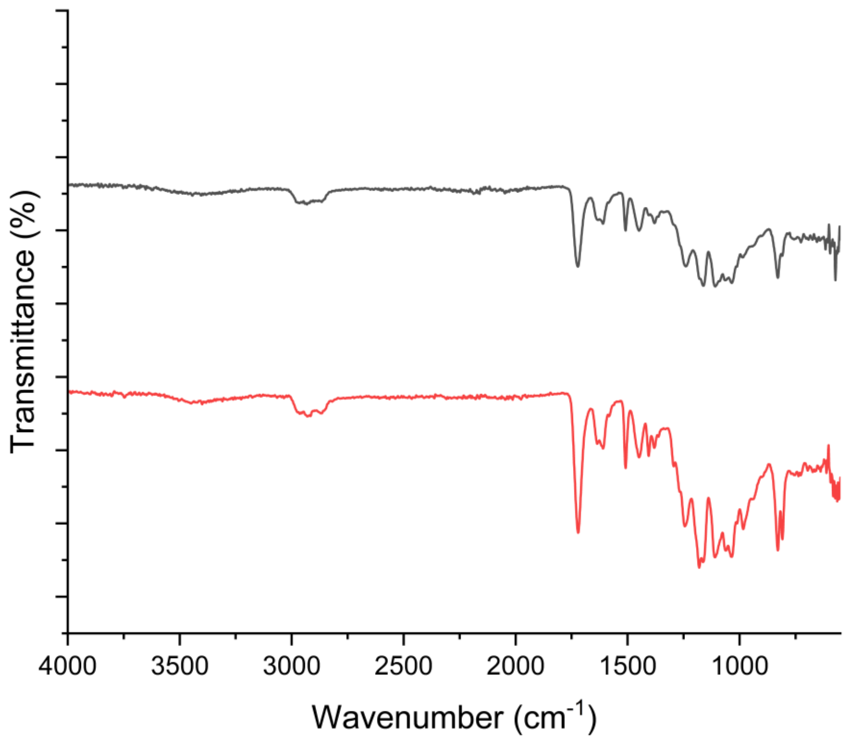

Figure S25. IR spectra of the $[Cu_3I_5(bz\text{-}ted)_2]$@3D0.24%, (black) and Standard Photopolymer Translucid Resin (red).

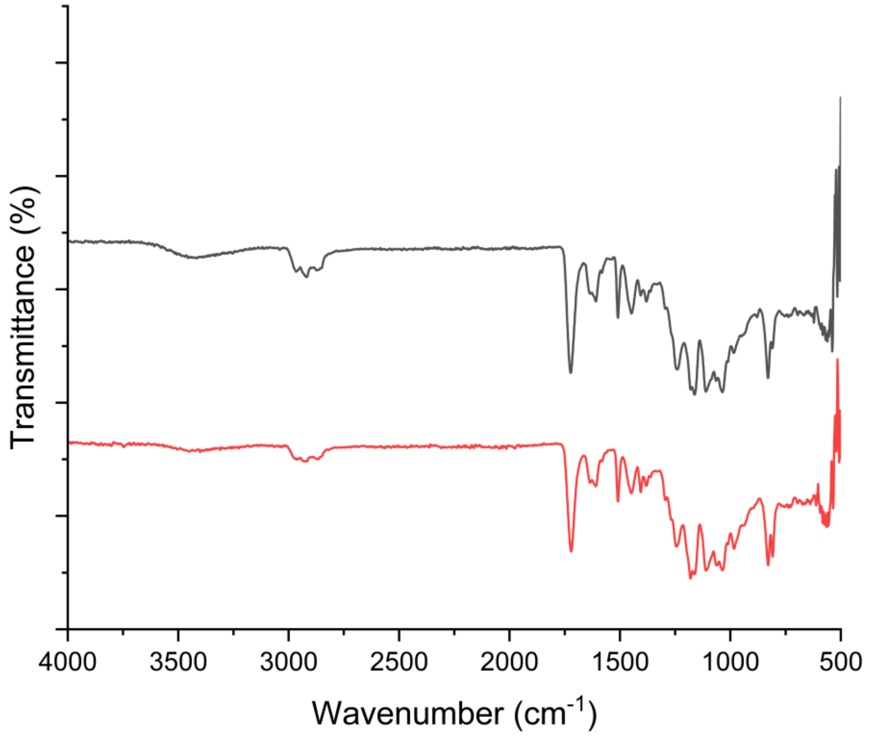

Figure S26. IR spectra of $[Cu_4I_6(pr\text{-}ted)_2]$@3D0.1% (black) and Standard Photopolymer Translucid Resin (red).

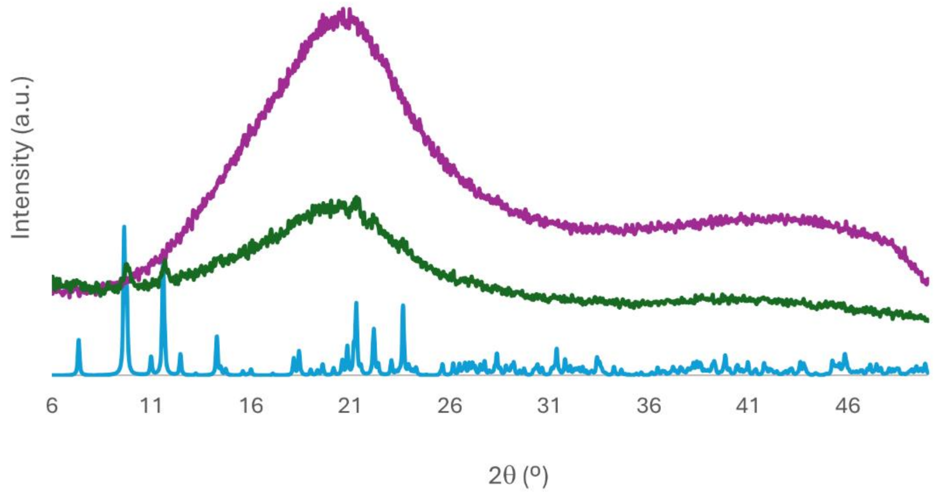

Figure S27. PXRD of the obtained $[Cu_3I_5(bz\text{-}ted)_2]$ (blue), $[Cu_3I_5(bz\text{-}ted)_2]$@3D0.24% (green) and Standard Photopolymer Translucid Resin (purple).

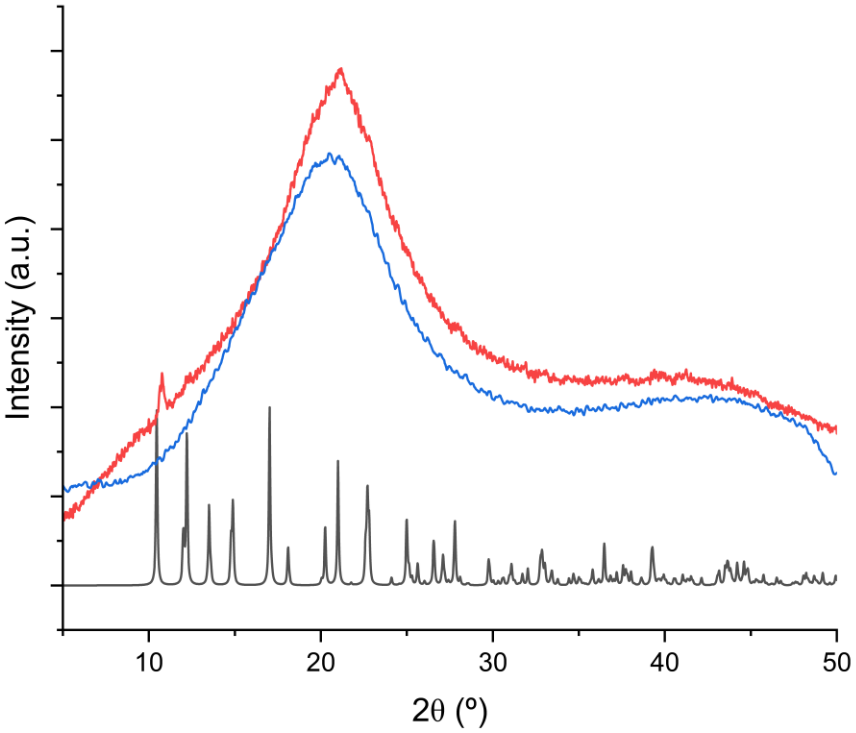

Figure S28. XRD theoretical pattern of [Cu_4I_6(pr-ted)$_2$] (black), PXRD of [Cu_4I_6(pr-ted)$_2$]@3D0.24% (red) and Standard Photopolymer Translucid Resin (blue).

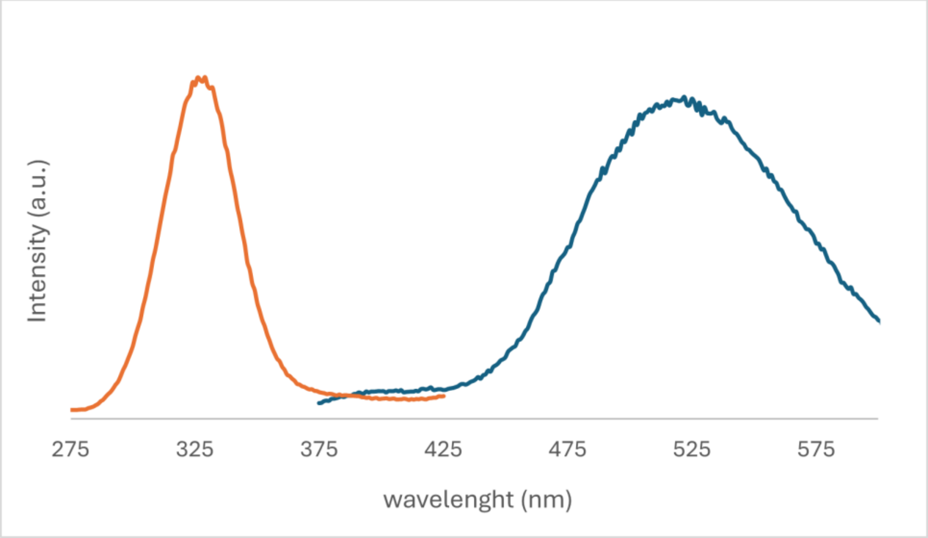

Figure S29. [Cu_3I_5(bz-ted)$_2$]@3D0.24% excitation spectrum (orange) (λ_{em}= 520 nm) and [Cu_3I_5(bz-ted)$_2$]@3D0.24% emission spectrum (blue) (λ_{ex}= 326 nm) in solid state.

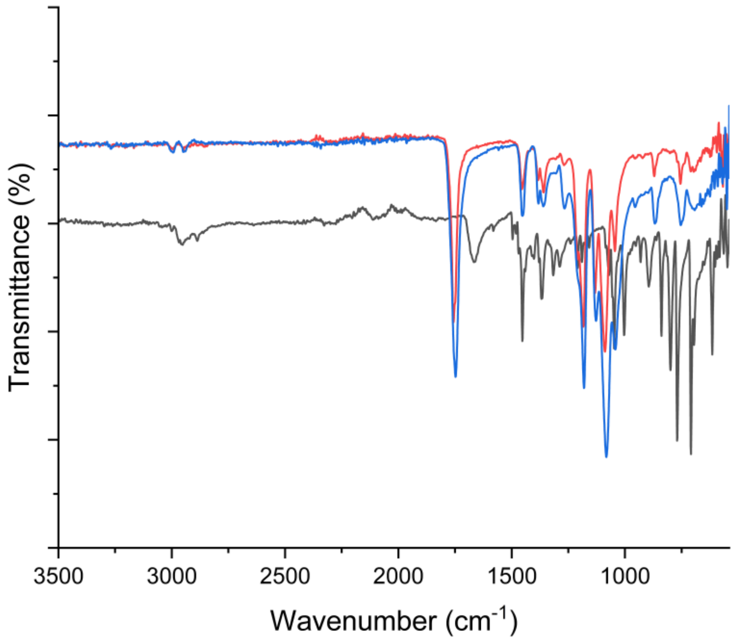

Figure S30. FT-IR spectra of [Cu_3I_5(bz-ted)$_2$] (black), [Cu_3I_5(bz-ted)$_2$]@PLA3,6% (red) and PLA (blue).

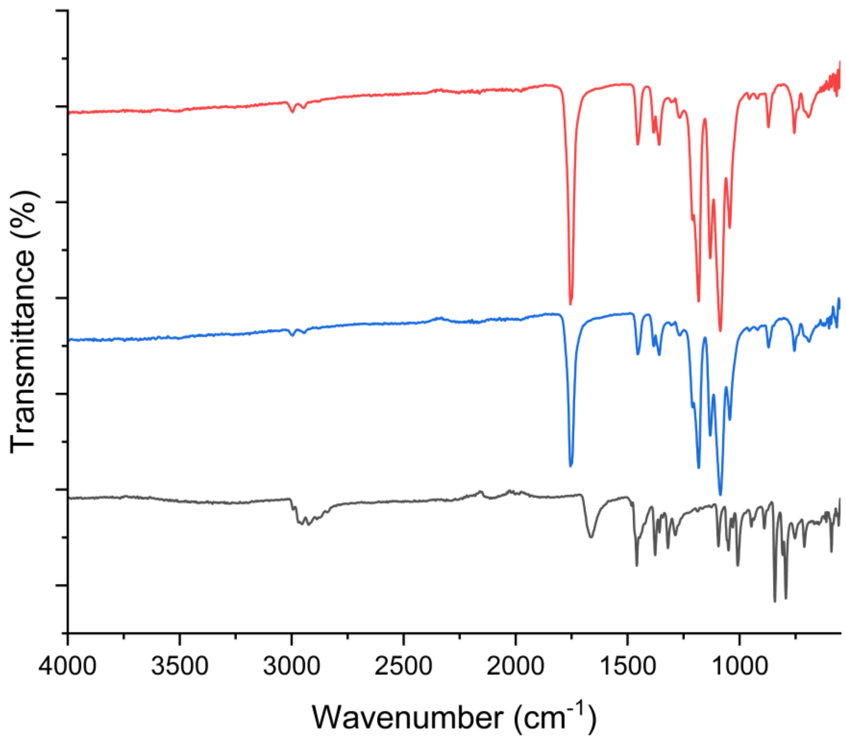

Figure S31. FT-IR spectra of [Cu_4I_6(pr-ted)$_2$] (black), [Cu_4I_6(pr-ted)$_2$]@PLA3.6% (red) and PLA (blue).

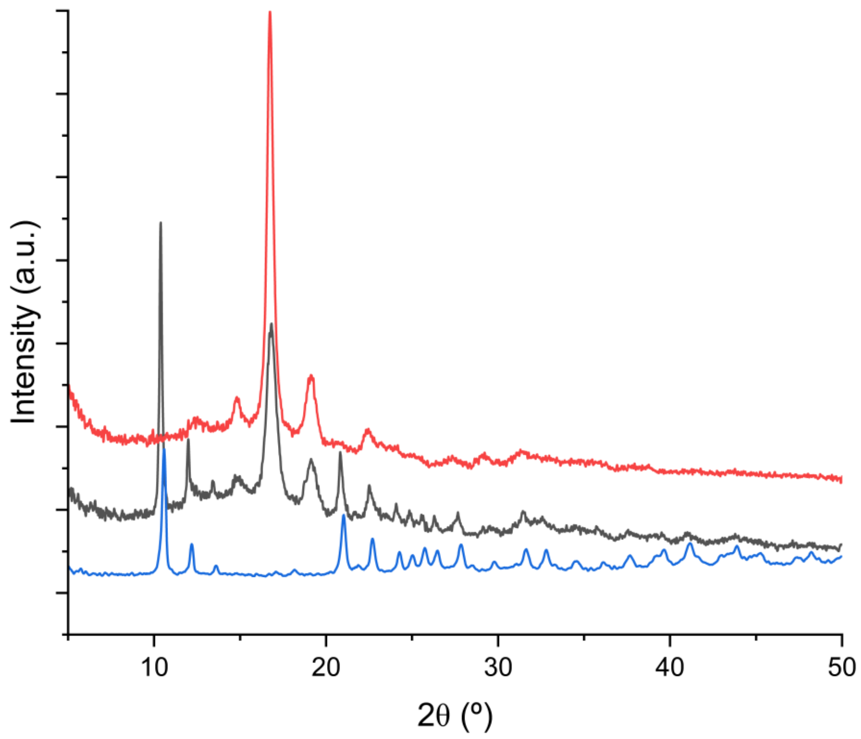

Figure S32. PXRD of $[Cu_4I_6(pr\text{-}ted)_2]$ (blue), $[Cu_4I_6(pr\text{-}ted)_2]$@PLA3.6% (black) and PLA in acetonitrile film (red).

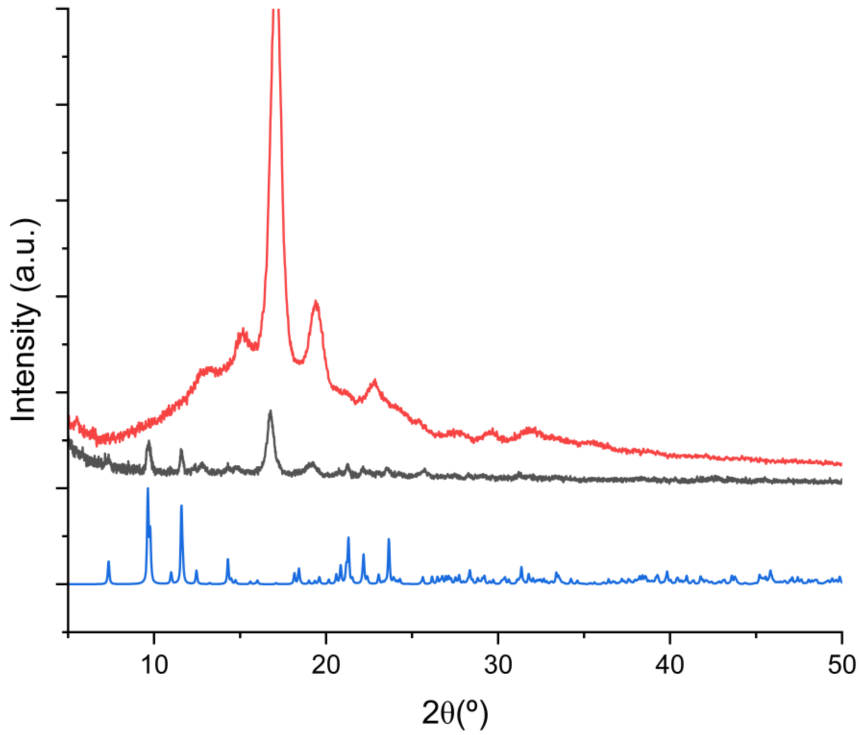

Figure S33. PXRD of $[Cu_3I_5(bz\text{-}ted)_2]$ (blue), $[Cu_3I_5(bz\text{-}ted)_2]$@PLA3.6% (black) and PLA (red) .

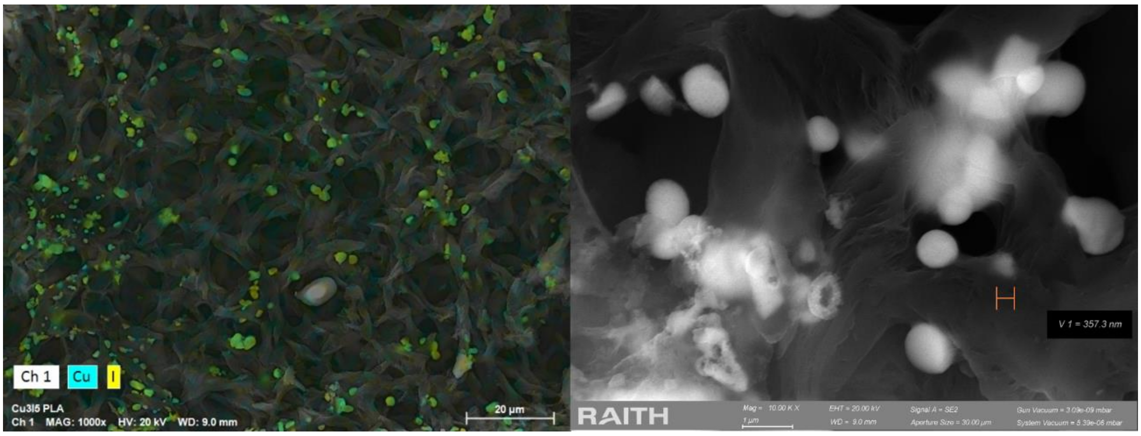

Figure S34. SEM-EDX map of $[Cu_3I_5(bz\text{-}ted)_2]$ distribution in the PLA film ($[Cu_3I_5(bz\text{-}ted)_2]$@PLA3.6%) (left image) and SEM image of a specific region of the $[Cu_3I_5(bz\text{-}ted)_2]$@PLA3.6% composite (right image).

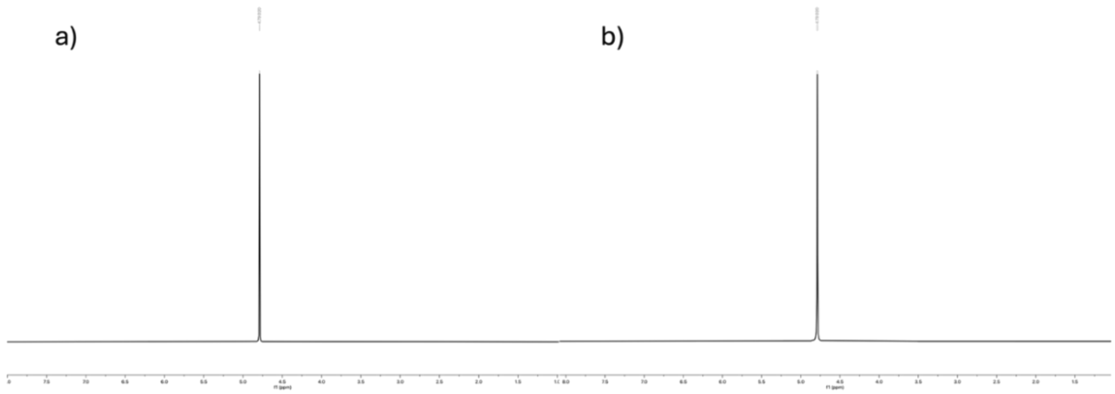

Figure S35. ^{1}H-RMN spectrum of $[Cu_3I_5(bz\text{-}ted)_2]$@3D0.24% in D_2O at t_0=0 (a), and after 2 hours (b).

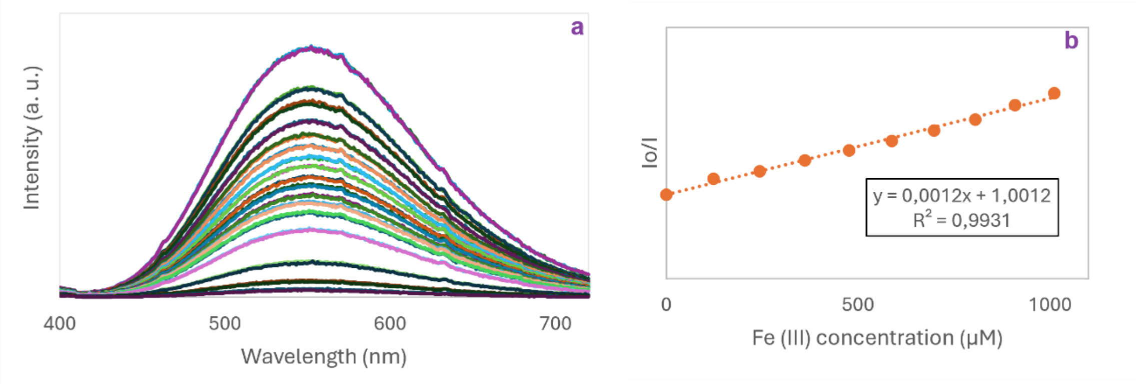

Figure S36. a) Emission spectra of an aqueous suspension of submicrometric particles of $[Cu_3I_5(bz\text{-}ted)_2]$ with respect to the Fe(III) concentration in deionized water, b) S-V diagram for the quenching of $[Cu_3I_5(bz\text{-}ted)_2]$ in deionized water in the presence of Fe (III) in the concentration range 0 - 1000 µM

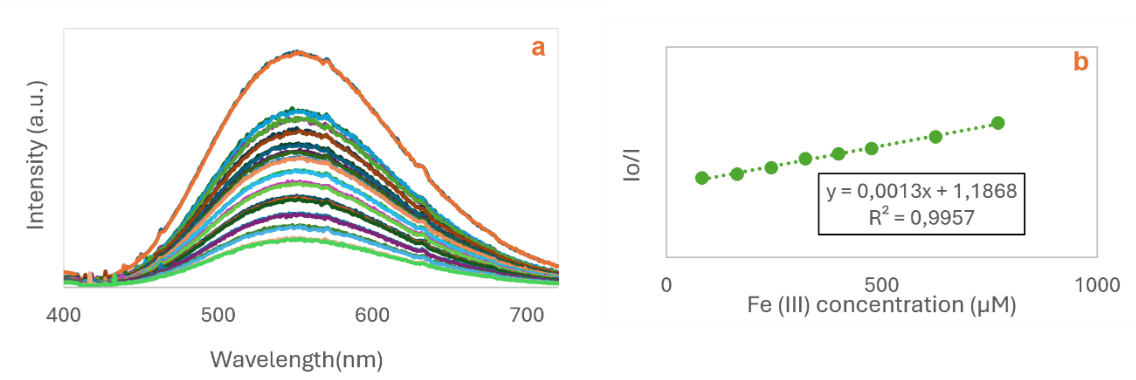

Figure S37. Emission spectra of an aqueous suspension of submicrometric particles of $[Cu_3I_5(bz\text{-}ted)_2]$ with respect to the Fe(III) concentration in river water, b) b) S-V diagram

for the quenching of $[Cu_3I_5(bz\text{-}ted)_2]$ in river water in the presence of Fe (III) in the concentration range 0 - 900 µM

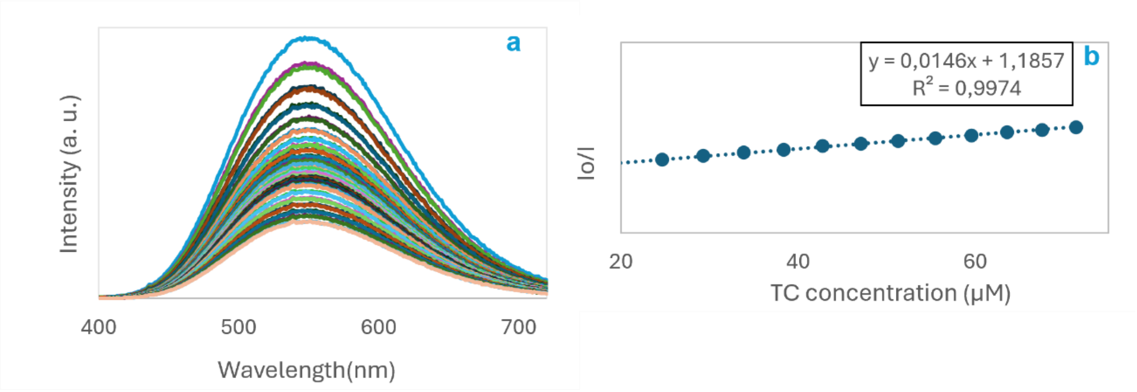

Figure S38. Emission spectra of an aqueous suspension of submicrometric particles of $[Cu_3I_5(bz\text{-}ted)_2]$ with respect to the TC concentration in river water, b) S-V diagram for the quenching of $[Cu_3I_5(bz\text{-}ted)_2]$ in river water in the presence of TC in the concentration range 20 - 70µM

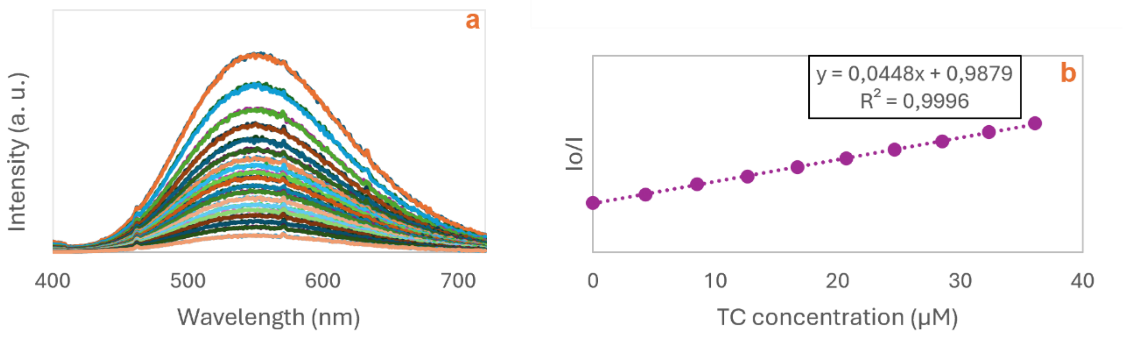

Figure S39. a) Emission spectra of an aqueous suspension of submicrometric particles of $[Cu_3I_5(bz\text{-}ted)_2]$ with respect to the TC concentration in deionized water, b) S-V diagram for the quenching of $[Cu_3I_5(bz\text{-}ted)_2]$ in deionized water in the presence of TC in the concentration range 20 - 70µM.

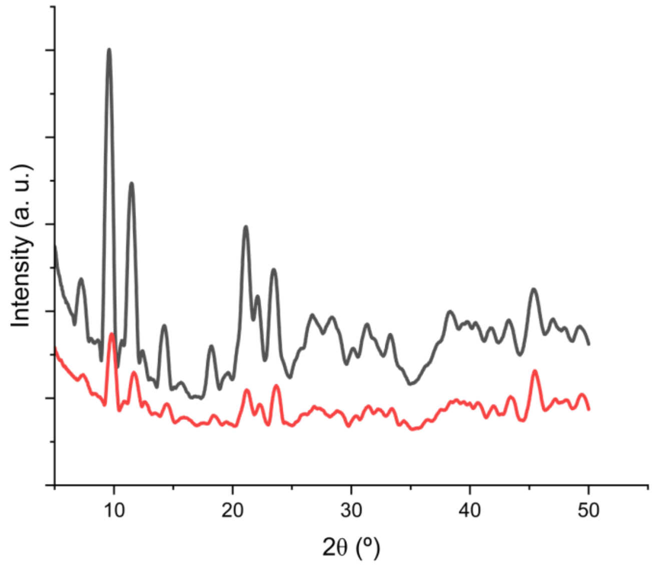

Figure S40. $[Cu_3I_5(bz\text{-}ted)_2]$ before (red) and after (black) interacting with Fe(III).

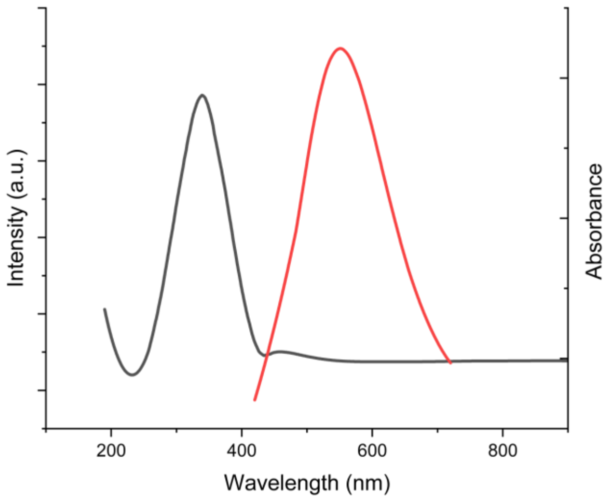

Figure S41. $[Cu_3I_5(bz\text{-}ted)_2]$ emission at room temperature (red) and $Fe(NO_3)_3$ absorbance (black).

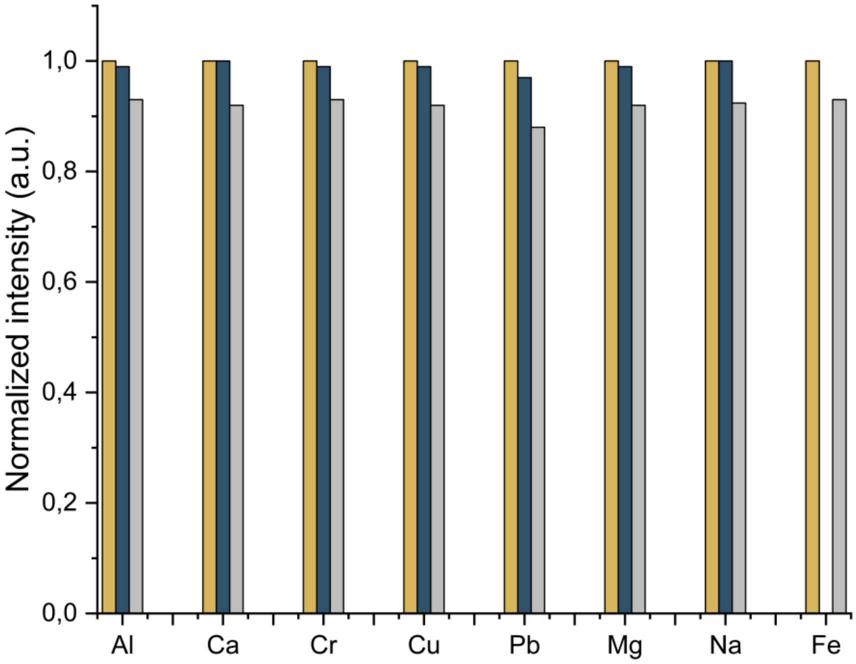

Figure S42. Study of the interference of $[Cu_3I_5(bz\text{-}ted)_2]$ with different metal ions for the detection of Fe(III). Pristine emission (yellow), emission after addition of 200 µL of a 0.01 M aqueous solution of the interfering metal ion (dark blue), and emission after addition of 200 µL of a 0.01 M aqueous solution of Fe(III) (grey).

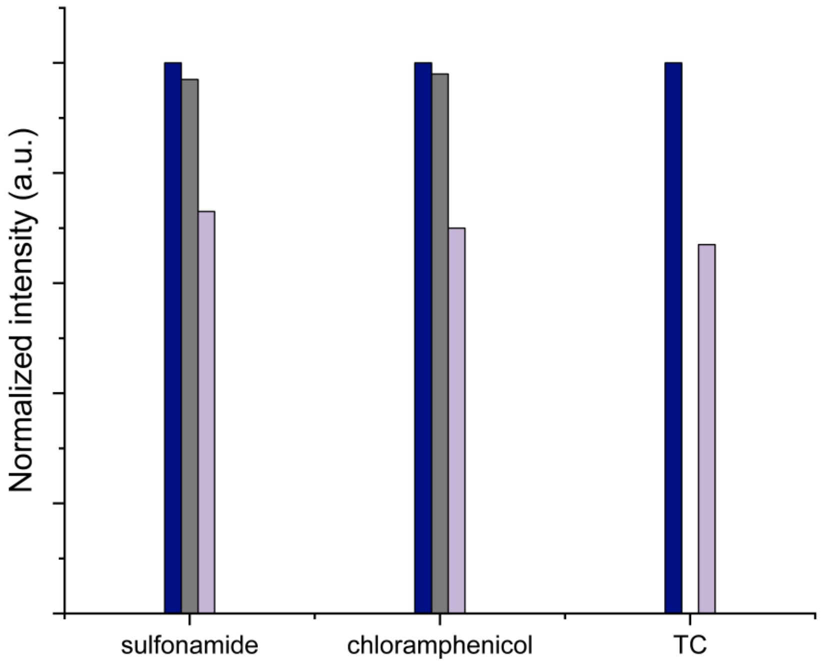

Figure S43. Study of interference by antibiotics on the emission of [Cu_3I_5(bz-ted)$_2$]: pristine emission (dark blue), emission after addition of 30 µL of a 10^{-3} M aqueous solution of the interfering antibiotic (dark grey), and emission after addition of 300 µL of a 10^{-4} M aqueous solution of TC (grey).

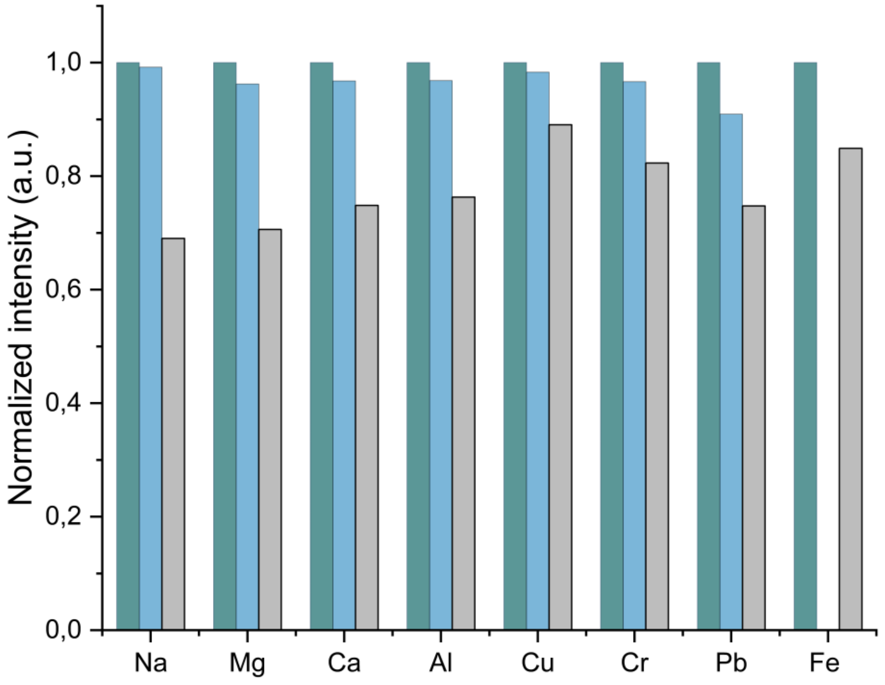

Figure S44. Study of interference by metal ions on the emission of [Cu_4I_6(pr-ted)$_2$]: pristine emission (light green), emission after addition of 200 μL of a 0.01 M aqueous solution of the interfering metal ion (light blue), and emission after addition of 200 μL of a 0.01 M aqueous solution of Fe(III) (grey).

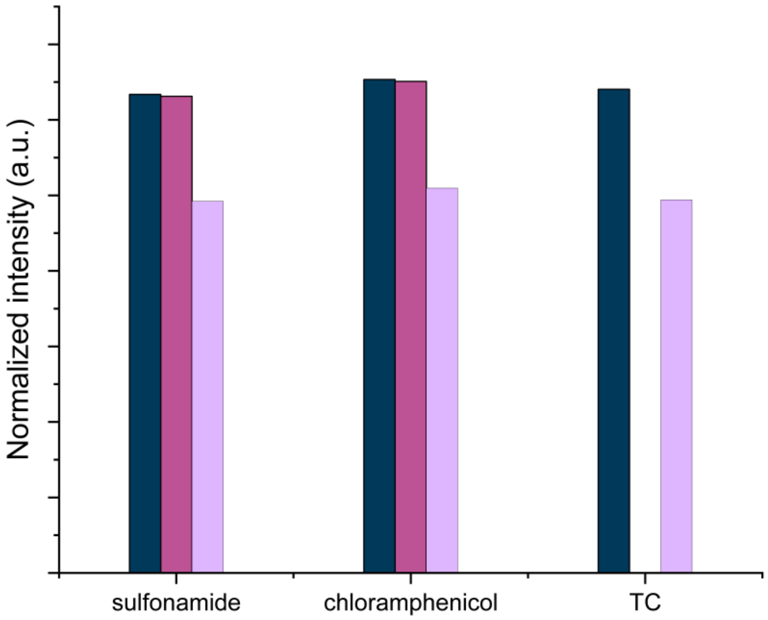

Figure S45. Study of interference by antibiotics on the emission of [Cu_4I_6(pr-ted)$_2$]: pristine emission (dark blue), emission after addition of 30 µL of a 10^{-3} M aqueous solution of the interfering antibiotic (dark...), and emission after addition of 300 µL of a 10^-4 M aqueous solution of TC (purple).

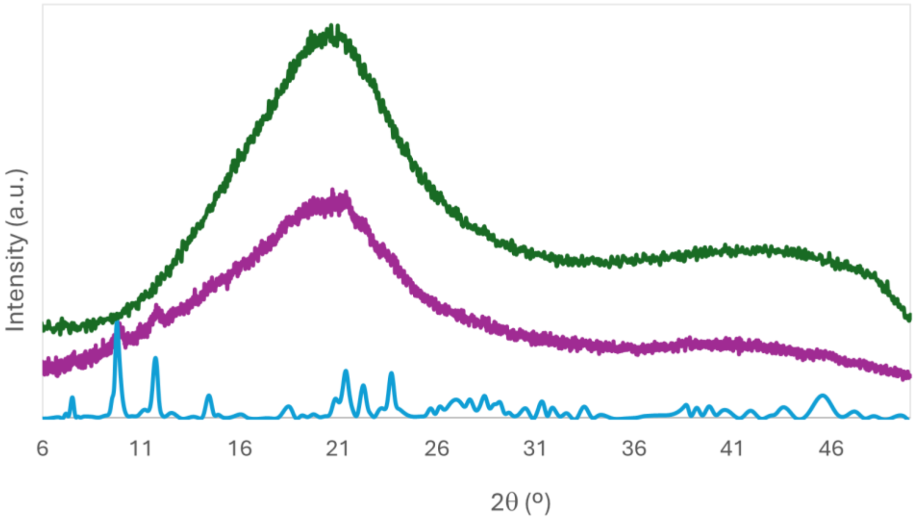

Figure S46. PXRD patterns of [Cu_3I_5(bz-ted)$_2$]@3D0.24%, after 20 cycles of **Fe (III) detection**. Simulated [Cu_3I_5(bz-ted)$_2$] (blue), [Cu_3I_5(bz-ted)$_2$]@3D$_{0\cdot24}$% (purple), and Standard Photopolymer Translucid Resin (green).

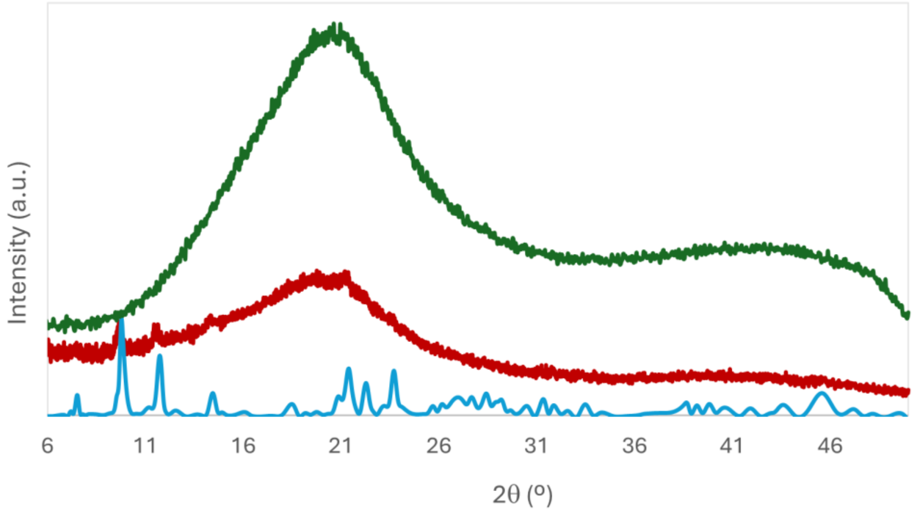

Figure S47. PXRD patterns of $[Cu_3I_5(bz\text{-}ted)_2]$@3D0.24%, after 20 cycles of **TC detection**. Simulated $[Cu_3I_5(bz\text{-}ted)_2]$ (blue), $[Cu_3I_5(bz\text{-}ted)_2]$@$3D_{0.24}$% (purple), and Standard Photopolymer Translucid Resin (green).
